# Balancing Brightness and Photobasicity: Modulating Excited-State Proton Transfer Pathways in Push–Pull Fluorophores for Biological Two-Photon Imaging

**DOI:** 10.1021/acs.jpca.4c05649

**Published:** 2024-11-07

**Authors:** Adam M. McCallum, Jiyao Yu, S. Sumalekshmy, Abigail Hagwood, Christoph J. Fahrni

**Affiliations:** School of Chemistry and Biochemistry and Petit Institute for Bioengineering and Bioscience, Georgia Institute of Technology, 901 Atlantic Drive, Atlanta, Georgia 30332, United States

## Abstract

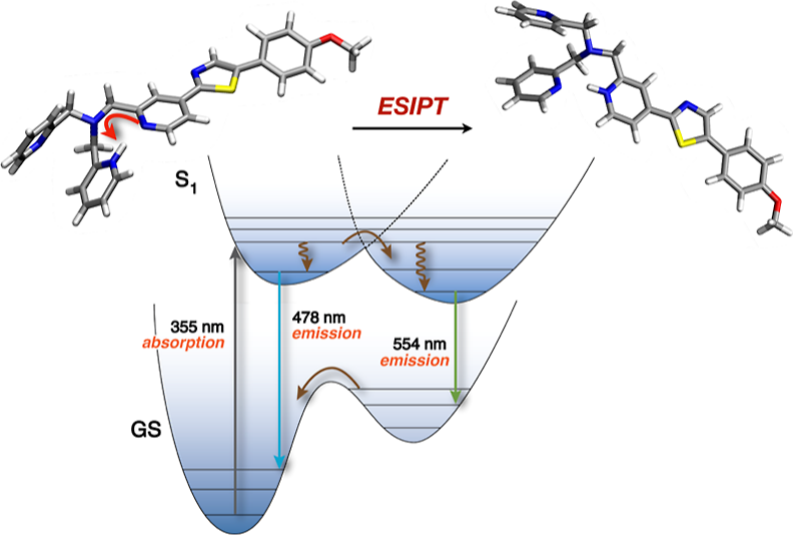

Push–pull fluorophores with donor–π–acceptor architectures are attractive scaffolds for the design of probes and labels for two-photon microscopy. Such fluorophores undergo a significant charge-delocalization in the excited state, which is essential for achieving a large two-photon absorption cross-section and brightness. The polarized excited state may, however, also facilitate excited-state proton transfer (ESPT) pathways that can interfere with the probe response. Herein, we employed steady-state and time-resolved spectroscopic studies to elucidate whether ESPT is responsible for the pH-dependent emission response of the Zn(II)-selective fluorescent probe chromis-1. Composed of a push–pull architecture with a pyridine ring as the acceptor, the chromis-1 fluorophore core acts as a photobase that promotes ESPT upon acidification. Although the p*K*_a_ of the pyridine acceptor increases more than six orders of magnitude upon excitation, the photobasicity is not sufficient to deprotonate solvent water molecules under neutral conditions. Rather, the pH-dependent emission response is caused by the pendant bis-isonicotinic acid chelating group which upon protonation facilitates an excited-state intramolecular proton transfer to the pyridine acceptor. A simple permutation of the core pyridine nitrogen from the para- to the ortho-position relative to the thiazole substituent was sufficient to reduce the excited-state basicity by two orders of magnitude without compromising the two-photon excited brightness. These results highlight the importance of choosing the appropriate fluorophore core and chelating moiety for minimizing pH-dependent responses in the design of fluorescent probes for biological imaging.

## Introduction

**Chart 1:**
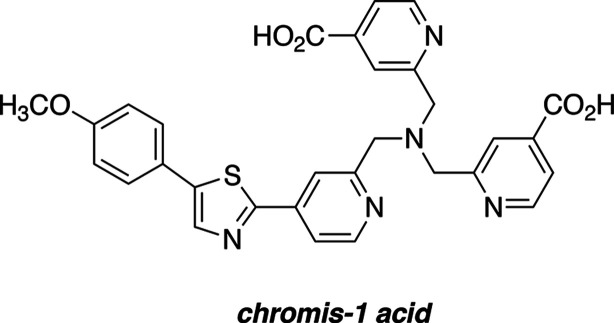


Two-photon excitation microscopy (TPEM) has emerged as an indispensable tool in biological research for deep-tissue and intravital imaging.^1−4^ Compared to traditional confocal fluorescence microscopy, TPEM relies on the simultaneous absorption of two low-energy photons in the near-infrared region, which increases the penetration depth and minimizes tissue scattering, background autofluorescence, and phototoxicity. Although traditional fluorescent probes and tags can be employed in TPEM, their brightness is often lower due to a less favorable absorption cross-section compared to one-photon excitation. According to established structure–activity relationships, dipolar and quadrupolar fluorophores composed of a donor–acceptor substituted π-system typically yield large two-photon absorption cross sections.^5,6^ When combined with a high quantum yield, such fluorophores provide high sensitivity in TPEM imaging for the detection of a wide range of analytes.^7−11^ Employing such a push–pull fluorophore architecture, we designed the Zn(II)-selective fluorescent probe, chromis-1 (Chart 1), which exhibits a two-photon absorption cross-section of around 20 GM and responds with a bathochromic emission shift upon Zn(II)-binding suitable for ratiometric image analysis.^12^ With a Zn(II) dissociation constant in the low nanomolar range, chromis-1 is well-suited for visualizing changes of buffered Zn(II) levels in live cells.^13^ For example, ratiometric image analysis revealed significant alterations in labile Zn(II) in oligodendrocytes upon their differentiation and maturation.^12,14^

In addition to responding with high selectivity toward the target metal ion, the emission of fluorescent probes should remain unaffected by changes in pH. As some subcellular compartments may be considerably acidic, additional control experiments should be performed to test for the interference of protonation-induced emission shifts. This can be accomplished, for example, by equilibrating the cellular pH with a proton pump inhibitor such as bafilomycin A1.^12,15^ Despite its utility in live-cell TPEM, the emission maximum of chromis-1 undergoes a distinct bathochromic shift upon acidification to pH 5 in the absence of Zn(II). Although the fluorescence intensity at acidic pH is significantly weaker compared to the Zn(II)-bound probe, the emission shift may still impact the ratiometric image analysis.

An apparent explanation for the pH-sensitivity of chromis-1 is the protonation of the pyridine nitrogen in the fluorophore core; however, UV–vis spectrophotometric titrations revealed that the two isonicotinic acid moieties are substantially more basic than the central pyridine ring, which is protonated only under strongly acidic conditions below pH 2. We therefore suspected that the pH-dependent fluorescence emission shift is due to an excited-state proton transfer (ESPT) process, which might even occur under mildly acidic conditions. Upon excitation, the push–pull fluorophore architecture of chromis-1 yields a polarized charge-transfer state with an increased electron density on the core pyridine nitrogen, which might facilitate excited-state protonation. Thus, the undesired pH-induced response of chromis-1 provided an impetus to evaluate the intrinsic photobasicity of the fluorophore core and to elucidate the role of the pendant isonicotinic acid moieties in assisting the ESPT in aqueous buffer. Detailed steady-state and time-resolved fluorescence studies revealed that the fluorophore core of chromis-1 is indeed a sufficiently strong photobase to elicit an intramolecular excited-state proton transfer (ESIPT) from the protonated isonicotinic acid moiety at mildly acidic pH, whereas intermolecular ESPT from neutral solvent water molecules constitutes only a minor pathway. Moreover, a simple structural change of the fluorophore architecture was sufficient to mitigate the ESPT reaction without compromising the two-photon-excited brightness. These results highlight the importance of the pendant chelating moiety in probe design, not only for achieving the desired metal ion selectivity and affinity but also for minimizing potential pH-dependent responses.

## Experimental Section

### Synthesis Reagents and Methods

Chromis-1 acid and 2-amino-4′-methoxyacetophenone **2** were synthesized according to previously published procedures.^12^ Commercially available isonicotinic acid (Eastman) and the Lawesson’s reagent (Sigma-Aldrich) were used without further purification. Flash chromatography purification was performed on silica gel (60 Å pore size, 250 mesh, Sorbent Technologies). ^1^H NMR spectra were recorded at 400 MHz at ambient temperature and referenced to TMS (0 ppm) for all solvents except D_2_O, which was referenced to externally added sodium 3-trimethylsilylpropionate-2,2,3,3-*d*_6_ (0 ppm). ^13^C NMR spectra were acquired at 100 MHz and referenced to the known chemical shift of the solvent peak (CDCl_3_: 77.0 ppm; DMSO-*d*_6_: 39.5 ppm; CD_3_OD: 49.0 ppm), excluding D_2_O, which was referenced to sodium 3-trimethylsilylpropionate-2,2,3,3-*d*_6_ (0 ppm). Mass spectra were recorded by the Georgia Tech Mass Spectrometry Facility.

### *N*-(2-(4-Methoxyphenyl)-2-oxoethyl)isonicotinamide (**3**)

To a solution of 2-amino-4′-methoxyacetophenone hydrochloride **2** (1.1 eq., 4.96 mmol, 1.00 g), isonicotinic acid (1 eq., 4.51 mmol, 555 mg), EDCI·HCl (1.5 eq., 6.77 mmol, 1.30 g), and HOBt (0.5 eq., 2.26 mmol, 305 mg) in DMF (15 mL), pyridine (1 eq., 4.51 mmol, 0.36 mL) was added via a syringe, and the resulting mixture was stirred overnight at room temperature. After slow addition of diH_2_O (60 mL), the product crystallized, and the colorless crystalline material was collected by filtration, washed with cold diH_2_O, and dried under vacuum overnight. Yield: 1.01 g (3.75 mmol, 83%). ^1^H NMR (CDCl_3_, 400 MHz) δ: 3.91 (s, 3H), 4.90 (d, *J* = 4.2 Hz, 2H), 7.01 (d, *J* = 9.0 Hz), 7.49 (s, 1H), 7.73 (dd, *J* = 4.5, 1.5 Hz, 2H), 8.01 (d, *J* = 9.0 Hz, 2H), 8.79 (d, *J* = 4.9 Hz, 2H). ^13^C NMR (CDCl_3_, 100 MHz) δ: 46.3, 55.5, 114.1, 121.0, 127.0, 130.3, 141.0, 150.5, 163.4, 165.3, 192.0. EI-MS *m*/*z* 270 ([M]^+^, 5%), 241 (45%), 135 (100%), 77 (20%). EI-HRMS *m*/*z* calculated for C_15_H_14_N_2_O_3_, 270.0999; found, 270.1004.

### 5-(4-Methoxyphenyl)-2-(pyridin-4-yl)thiazole (**1a**)

Iso-nicotinamide derivative **3** (1 eq., 3.36 mmol, 908 mg) and Lawesson’s reagent (1.3 eq., 4.37 mmol, 1.77 g) were placed in an oven-dried 2-neck 100 mL round-bottom flask equipped with a stir bar, reflux condenser, and bubbler. After flushing with argon, butyronitrile (15 mL, dried over 4 Å molecular sieves) was added, and the mixture was stirred in an oil bath at 140 °C for 30 min. After cooling to room temperature, 2 mL of saturated aq. NaHCO_3_ was added. The deep-red mixture was diluted with ethyl acetate (40 mL), transferred to a separatory funnel, and washed with additional 5% (w/v) aq. NaOH and brine. The organic layer was separated, dried over MgSO_4_, and concentrated under reduced pressure to afford a yellow solid residue. The crude product was recrystallized from a 1:1 mixture of 2,2,4-trimethylpentane and toluene to afford **1a** as a pale-yellow solid. Yield: 670 mg (2.50 mmol, 74%). ^1^H NMR (CDCl_3_, 400 MHz) δ: 3.83 (s, 3H), 6.94 (d, *J* = 8.8 Hz, 2H), 7.54 (d, *J* = 8.8 Hz, 2H) 7.77 (dd, *J* = 4.6, 1.6, 2H), 7.97 (s, 1H), 8.69 (dd, *J* = 4.6, 1.6 Hz, 2H). ^13^C NMR (CDCl_3_, 100 MHz) δ: 55.3, 114.6, 119.8, 123.2, 128.1, 138.8, 140.3, 141.3, 150.5, 160.1, 162.7. EI-MS *m*/*z* 268 ([M]^+^, 100%), 253 (40%), 164 (23%), 149 (43%), 121 (33%), 77 (25%). EI-HRMS *m*/*z* calculated for C_15_H_12_N_2_OS, 268.0665; found, 268.0670.

### 4-(2-(Pyridin-4-yl)thiazol-5-yl)phenol (**4**)

Compound **1a** (1 eq., 0.373 mmol, 100 mg) was added to a 10 mL round-bottom flask equipped with a stir bar. The flask was sealed with a rubber septum, evacuated under high vacuum, and backfilled with argon. Via an argon-filled syringe, anhydrous dichloromethane (2 mL) was transferred to the flask to dissolve the starting material. The flask was then lowered into an ice bath at 0 °C, and upon thermal equilibration (∼10 min), boron tribromide (3 eq., 1.12 mmol, 1 M solution in dichloromethane) was added dropwise to the stirred solution. After stirring at 0 °C for 6 h, the mixture was diluted with ethyl acetate (10 mL), quenched with diH_2_O (10 mL), and the pH was adjusted to ∼7 with saturated aq. NaHCO_3_. The aqueous layer was further extracted with ethyl acetate (2 × 10 mL), and the combined organic layers were dried over MgSO_4_, filtered, and concentrated to yield an orange solid. The crude material was recrystallized from methanol to afford **4** as an orange solid. Yield: 55 mg (0.216 mmol, 58%). ^1^H NMR (DMSO-*d*_6_, 400 MHz) δ: 6.88 (d, *J* = 8.6 Hz, 2H), 7.58 (d, *J* = 8.6 Hz, 2H), 7.86 (dd, *J* = 4.6, 1.5 Hz, 2H), 8.27 (s, 1H), 8.71 (dd, *J* = 4.6, 1.5 Hz, 2H). 9.92 (s, 1H). ^13^C NMR (DMSO-*d*_6_, 100 MHz) δ: 116.1, 119.6, 121.1, 128.2, 138.9, 139.6, 141.5, 150.7, 158.4, 161.4. EI-MS *m*/*z* 254 ([M]^+^, 100%), 150 (73%), 121 (28%). EI-HRMS *m*/*z* calculated for C_14_H_10_N_2_OS, 254.0508; found, 254.0511.

### 3-(4-(2-(Pyridin-4-yl)thiazol-5-yl)phenoxy)propane-1-sulfonate Sodium Salt (**1b**)

In a 10 mL round-bottom flask equipped with stir bar, phenol derivative **4** (1 eq., 0.236 mmol, 60 mg) was suspended in anhydrous THF (5 mL) under argon. Sodium *tert*-butoxide (NaO^*t*^Bu, 1.0 eq., 0.236 mmol, 22.7 mg) was added to a separate 4 mL vial sealed with a septum, and the vial was evacuated and backfilled with argon. The NaO^*t*^Bu was suspended in anhydrous THF (2 mL), transferred to an argon-flushed syringe body, and the suspension was added dropwise to the stirred solution containing phenol **4**. After 30 min of stirring, 1,3-propane sultone (1.1 eq., 0.260 mmol, 32 mg) was added, and the solution was stirred vigorously overnight. The THF was removed under reduced pressure to yield a crude yellow solid, which was purified by column chromatography (silica gel, 4:1 dichloromethane-methanol) to afford sulfonate **1b** as a yellow crystalline solid. Yield: 76 mg (0.201 mmol, 81%). ^1^H NMR (DMSO-*d*_6_, 500 MHz) δ: 2.02–2.1 (m, 2H), 2.61 (t, *J* = 7.3 Hz, 2H), 4.17 (t, *J* = 6.6 Hz, 2H), 7.09 (d, *J* = 8.8 Hz, 2H), 7.73 (d, *J* = 8.8 Hz, 2H), 7.93 (dd, *J* = 4.5, 1.7 Hz, 2H), 8.38 (s, 1H), 8.75 (dd, *J* = 4.5, 1.6 Hz, 2H). ^13^C NMR (DMSO-*d*_6_, 100 MHz) δ: 25.2, 47.8, 66.9, 115.3, 119.7(2), 119.7(3), 128.1, 139.4(9), 139.5(6), 141.0, 150.7, 159.3, 161.8. ESI-HRMS *m*/*z* calculated for C_17_H_15_N_2_O_4_S_2_, 375.0479; found, 375.0478.

### *N*-(2-(4-Methoxyphenyl)-2-oxoethyl)picolinamide (**6**)

A 250 mL round-bottom flask equipped with a stir bar was charged with 2-amino-4′-methoxyacetophenone hydrochloride **2** (1.1 eq., 14.9 mmol, 3.0 g), picolinic acid (1 eq., 13.5 mmol, 1.66 g), EDCI·HCl (1.5 eq., 20.3 mmol, 3.89 g), and HOBt (0.5 eq., 6.76 mmol, 913 mg). After dissolving the contents in 40 mL of DMF, pyridine (1 eq., 13.5 mmol, 1.09 mL) was added to the reaction solution via a syringe, and the solution was allowed to stir overnight. The product was crystallized out of solution by the slow addition of 100 mL of diH_2_O. The product was filtered off, washed with cold diH_2_O, and dried under vacuum to afford **6** as a colorless crystalline solid. Yield: 3.48 g (12.9 mmol, 95%). ^1^H NMR (CDCl_3_, 400 MHz) δ: 3.88 (s, 3H), 4.92 (d, *J* = 4.76 Hz, 2H), 6.98 (d, *J* = 8.8 Hz, 2H), 7.45 (ddd, *J* = 7.6, 4.8, 1.2 Hz, 1H), 7.85 (td, *J* = 7.7, 1.0 Hz, 1H), 8.02 (d, *J* = 8.8 Hz, 1H), 8.20 (dt, *J* = 7.8, 1.0 Hz, 1H), 8.63 (ddd, *J* = 4.8, 1.7, 0.9 Hz, 1H), 8.97 (s, 1H). ^13^C NMR (CDCl_3_, 100 MHz) δ: 45.9, 55.5, 114.0, 112.1, 126.2, 127.6, 130.2, 137.1, 148.3, 149.6, 164.1, 164.5, 192.1. EI-MS *m*/*z* 270 ([M]^+^, 5%), 135 (100%), 78 (23%). EI-HRMS *m*/*z* calculated for C_15_H_14_N_2_O_3_ (M^+^), 270.0999; found, 270.1002.

### 5-(4-Methoxyphenyl)-2-(pyridin-2-yl)thiazole (**5a**)

To an oven-dried 2-neck 100 mL round-bottom flask equipped with a stir bar was added picolinamide intermediate **6** (1 eq., 6.66 mmol, 1.80 g) and Lawesson’s reagent (1.3 eq., 8.65 mmol, 3.50 g). The flask was connected to a reflux condenser and flushed with argon for several minutes to remove moisture. Butyronitrile (40 mL, dried over 4 Å molecular sieves) was added, and the reaction mixture was heated under reflux with stirring for 20 min. After cooling to room temperature, saturated aq. NaHCO_3_ (2 mL) was added. The resulting deep-red mixture was diluted with ethyl acetate (40 mL), transferred to a separatory funnel and washed with saturated NaHCO_3_ and brine. The organic layer was dried over MgSO_4_, filtered, and concentrated under reduced pressure to afford a yellow solid. The crude solid was purified via column chromatography (silica gel) using a 7:2:1 mixture of hexanes/dichloromethane/MTBE to afford **5a** as a yellow solid. Yield: 1.36 g (5.07 mmol, 76%). ^1^H NMR (CDCl_3_, 400 MHz) δ: 3.83 (s, 3H), 6.94 (d, *J* = 8.8, 2H), 7.29 (ddd, *J* = 7.7, 5.0, 1.3 Hz, 1H), 7.56 (d, *J* = 8.9 Hz, 2H), 7.78 (td, *J* = 7.8, 1.7 Hz, 1H), 7.97 (s, 1H), 8.16 (dt, *J* = 8.0, 1.0, 1H), 8.61 (ddd, *J* = 4.9, 1.6, 0.9, 1H). ^13^C NMR (CDCl_3_, 100 MHz) δ: 55.3, 114.5, 119.2, 124.0, 124.1, 127.9, 136.9, 138.5, 141.5, 149.4, 151.5, 159.8, 166.9. EI-MS *m*/*z* 268 (100%, [M]^+^), 253 (40%), 149 (25%), 121 (20%). EI-HRMS *m*/*z* calculated for C_15_H_12_N_2_OS (M^+^), 268.0665; found, 268.0668.

### 4-(2-(Pyridin-2-yl)thiazol-5-yl)phenol (**7**)

Compound **5a** (1 eq., 0.373 mmol, 100 mg) was added to a 10 mL round-bottom flask equipped with a stir bar. The flask was sealed with a rubber septum, evacuated and then backfilled with argon. After adding anhydrous dichloromethane (2 mL), the solution was cooled to −78 °C in a dry ice/acetone bath. Boron tribromide (1.12 mL, 1 M solution in dichloromethane) was added dropwise with stirring. After stirring for 5 min at −78 °C, the reaction mixture was warmed to room temperature and stirred for another 2.5 h. The mixture was diluted with ethyl acetate (10 mL) and washed with diH_2_O (10 mL) and saturated aq. NaHCO_3_. The aqueous layer was extracted with ethyl acetate (2 × 10 mL), and the combined organic layers were dried over MgSO_4_, filtered, and concentrated. The crude product was purified via column chromatography (silica gel) using 1:1 ethyl acetate/hexanes, followed by recrystallization from 1:1 toluene/cyclohexane to afford **7** as a yellow crystalline solid. Yield: 73 mg (0.287 mmol, 77%). ^1^H NMR (CD_3_OD, 400 MHz) δ: 6.86 (d, *J* = 8.8, 2H), 7.41 (ddd, *J* = 7.5, 4.9, 1.2 Hz, 1H), 7.53 (d, *J* = 8.9, 2H), 7.90 (ddd, *J* = 7.9, 7.6, 1.7 Hz, 1H), 8.00 (s, 1H), 8.13 (dt, *J* = 8.0, 1.1 Hz, 1H), 8.56 (ddd, *J* = 4.9, 1.7, 1.0 Hz, 1H). ^13^C NMR (CD_3_OD, 100 MHz) δ: 117.0, 120.3, 123.7, 125.7, 129.1, 138.6, 139.0, 143.7, 150.5, 152.4, 159.5, 167.7. EI-MS *m*/*z* 254 (100%, [M]^+^), 150 (45%), 121 (20%). EI-HRMS *m*/*z* calculated for C_14_H_10_N_2_OS (M^+^), 254.0508; found, 254.0513.

### 3-(4-(2-(Pyridin-2-yl)thiazol-5-yl)phenoxy)propane-1-sulfonate Sodium Salt (**5b**)

To a 10 mL round-bottom flask equipped with a stir bar was added phenol intermediate **7** (1 eq., 0.248 mmol, 63 mg) and 1,4-dioxane (2 mL). Under magnetic stirring, NaOH (75 μL of a 20% (w/v) aqueous solution) was added, resulting in precipitation of the phenolate sodium salt, which was redissolved by adding a few drops of water. After 15 min, 1,3-propane sultone (30.3 mg, 0.248 mmol) was added, and the solution was stirred vigorously for 4 h. The solvent was removed under reduced pressure, and the product was crystallized from a 3:1 isopropanol/methanol mixture to afford **5b** as a yellow crystalline solid. Yield: 72 mg (0.181 mmol, 73%). ^1^H NMR (DMSO-*d*_6_, 400 MHz) δ: 2.00–2.06 (m, 2H), 2.58 (t, *J* = 7.4 Hz, 2H), 4.13 (t, *J* = 6.6 Hz, 2H), 7.03 (d, *J* = 8.8 Hz, 2H), 7.49 (ddd, *J* = 7.5, 4.8, 1.2 Hz, 1H), 7.69 (d, *J* = 8.8 Hz, 2H), 7.97 (td, *J* = 7.7, 1.7 Hz, 1H), 8.13 (dt, *J* = 7.9, 1.0 Hz, 1H), 8.27 (s, 1H), 8.64 (ddd, *J* = 4.8, 1.6, 1.0 Hz, 1H). ^13^C NMR (DMSO-*d*_6_, 100 MHz) δ: 25.2, 47.8, 66.9, 115.2, 118.8, 123.1, 124.9, 127.9, 137.7, 139.2, 140.9, 149.7, 150.6, 159.1, 166.0. ESI-HRMS *m*/*z* calculated for C_17_H_15_N_2_O_4_S_2_ (M^–^), 375.0479; found, 375.0476.

### Potentiometry

All protonation constants were obtained from spectrophotometric potentiometric titrations using a combination double junction glass electrode. The electrode was calibrated at 25 °C by titrating 5 mM aqueous HCl in 0.1 M KCl (ionic background) with a strong base (0.1 M KOH, standardized solution, Sigma-Aldrich) using a water-jacketed, temperature-controlled titration vessel (Metrohm). From the experimental emf data, the end point, electrode potential, and slope were determined using Gran’s Method as implemented in the GLEE software package (p*K*_w_ = 13.78 for μ = 0.1 M KCl).^16^ The calibration procedure was performed prior to each titration, and the experimental electrode potential and slope that were used to calculate the experimental p[H] were derived as the average of the emf data from three independent titrations. To determine the p*K*_a_’s, a 10 μM solution of the compound (diluted from a 3 mM stock solution in diH_2_O) was prepared in 3.0 mL of aqueous buffer (5 mM PIPBS, 0.1 M KCl, 5 mM KOH, 25 °C, pH 5.6) in a quartz cuvette with 1 cm path length. A combined potentiometric and spectrophotometric titration was carried out in the cuvette by incremental addition of HCl of various aliquot sizes and concentrations to adjust the pH across the target window (thermostat accessory set to 25 °C). After the addition of each aliquot of acid, the solution was equilibrated by magnetic stirring until the pH electrode stabilized, the potential was recorded (in mV), and an absorbance spectrum was acquired over the spectral window of 250–500 nm. After correcting for dilution, the absorbance data and corresponding potentials were analyzed by nonlinear least-squares fitting to a single protonation equilibrium model using the SPECFIT software.^17^

### UV–Vis and Fluorescence Spectroscopy

All buffers and stock solutions used for spectroscopic measurements were prepared using either HPLC-grade water (JT Baker) or 18.2 MΩ cm Milli-Q water and filtered through a 0.2 μm filter to remove interfering dust particles or fibers. UV–vis measurements were performed on a Varian Cary Bio50 spectrophotometer with a constant temperature accessory (Peltier) at 25 °C. Fluorescence measurements were acquired with a 1 cm path length quartz cuvette using a PTI fluorimeter equipped with a 75 W xenon arc lamp excitation source and a photomultiplier detection system (PMT voltage was 1100 V for all experiments). Fluorescence spectra were corrected for the spectral response of the detection system and for the spectral irradiance of the excitation source via a calibrated photodiode. Fluorescence quantum yields were determined in aqueous buffer (10 mM PIPES, 0.1 M KCl) at pH 7.0 by measuring the integrated fluorescence intensity with excitation at 365 nm of solutions with four distinct optical densities varying between 0.1 and 0.4 (using a 10 cm path length UV–vis cuvette). All fluorescence quantum yields were referenced to quinine sulfate (Φ_F_ = 0.546 in 1.0 N H_2_SO_4_) as the standard.^18^

### Quantum Chemical Calculations

All quantum chemical calculations were performed with the Gaussian 09 suite of programs (Rev. C01). Ground-state geometries were energy-minimized by DFT using the B3LYP functional and Pople’s 6-31+G(d) split valence basis set with added diffuse and polarization functions. All final geometries were verified by a frequency analysis to ensure a stationary point on the ground-state potential surface. Vertical excitation energies were estimated based on TD-DFT calculations using the long-range corrected CAM-B3LYP functional^19^ combined with the 6-31+G(d) basis set with added diffuse functions. To account for bulk solvent effects, the geometry optimizations were also performed with the polarizable continuum model (PCM)^20^ using default parameters followed by TD-DFT calculations with PCM equilibrium solvation. Fluorescence emission energies were estimated based on TD-DFT energy-minimized excited-state geometries with and without PCM equilibrium solvation at the CAM-B3LYP/6-31+G(d) level of theory. Orbital densities, electron density difference maps, and electrostatic potential surfaces were visualized with VMD based on the Gaussian cube output files.^21^

### TPEM

3T3 mouse fibroblasts were cultured in MaTek glass bottom Petri dishes using Dulbecco’s Modified Eagle Medium (DMEM) supplemented with 10% bovine calf serum, penicillin/streptomycin (50 IU/mL), and l-glutamine (200 μM). The growth medium was replaced with serum-free DMEM containing 2 μM of the fluorophore **1a** or **5a**, and the cells were incubated at 37 °C for 30 min (5% CO_2_ atmosphere). Cells were imaged with a Zeiss confocal NLO 710 microscope equipped with a femtosecond-pulsed Ti/sapphire laser with excitation at 720 nm (1% laser power). The fluorescence emission was collected from 425–705 nm with 16 bit (PMT) resolution. The average fluorescence intensity of individual cells (*n* = 100) was evaluated with ImageJ2 software.^22^

## Results and Discussion

### Protonation Equilibria of Chromis-1 Acid

**Figure 1:**
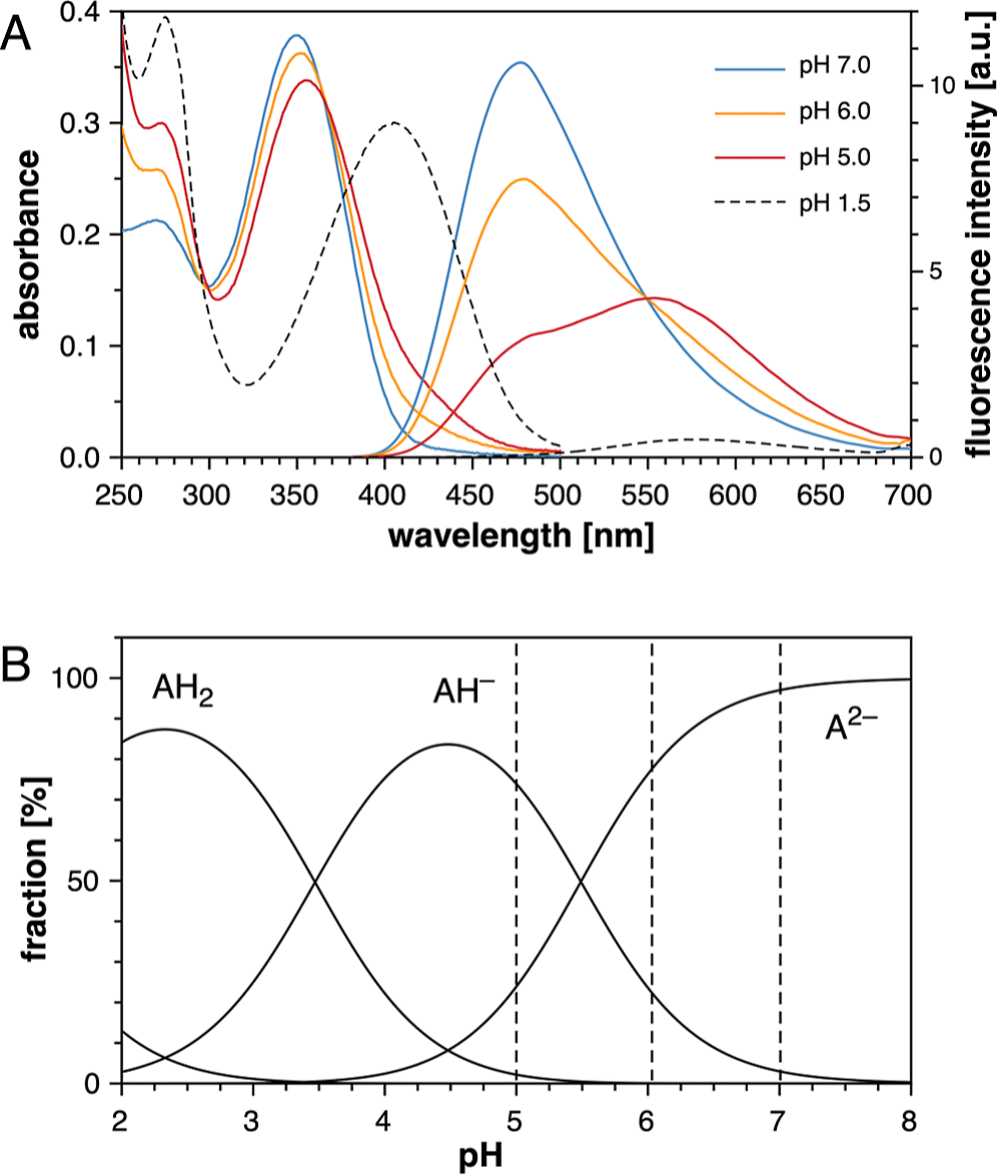
(A) UV–vis absorption (left) and fluorescence emission spectra (right) of chromis-1 acid in aqueous solution (0.1 M KCl) at pH 7.0 (blue), 6.0 (orange), 5.0 (red), and 1.5 (dashed trace). (B) Species distribution diagram of chromis-1 acid as a function of pH (A^2–^ refers to the fully deprotonated conjugate acid of chromis-1).

Previous potentiometric studies of chromis-1 acid revealed p*K*_a_’s of 5.5, 3.5, and 1.2 (0.1 M KCl, 1 mM PIPES/PIPBS, 25 °C).^12^ Although aliphatic tertiary amines are usually more basic than pyridine, the tertiary aliphatic nitrogen in tris(picolyl)amine is significantly less basic due to the inductive effects of the three electron-withdrawing picolyl substituents.^23^ Thus, the first p*K*_a_ of 5.5 of chromis-1 acid is likely associated with protonation of one of the pendant isonicotinic acid moieties. Factoring in the additional statistical contribution, this value agrees well with the p*K*_a_ of 4.94 for unsubstituted isonicotinic acid.^24^ Consistent with this assignment, the UV–vis spectrum of chromis-1 acid shows only a small bathochromic shift of the absorption maximum from 349 to 355 nm along with a slight decrease in intensity upon acidification to 5.0 (Figure 1A).

**Scheme 1:**
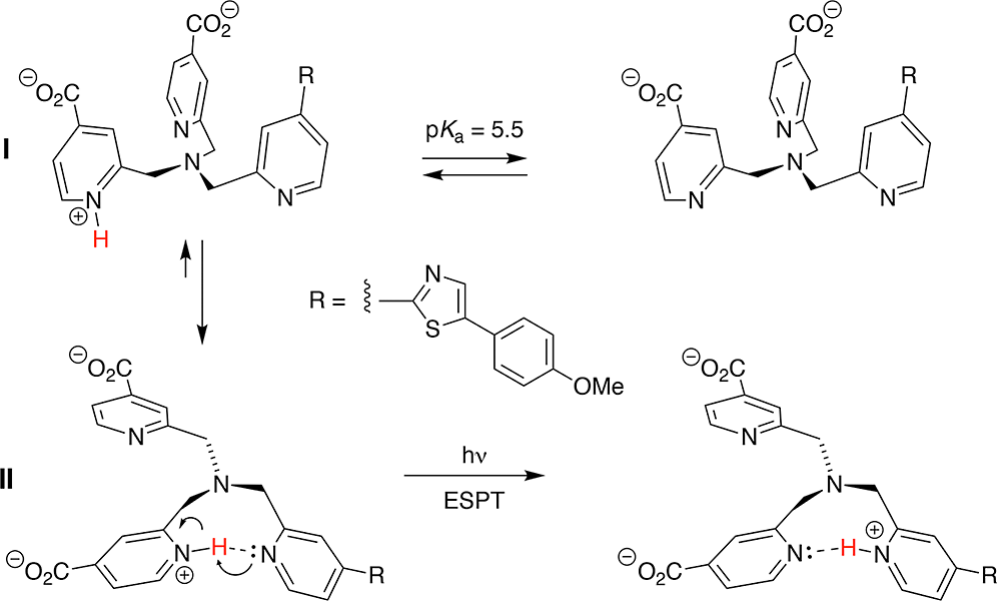


Unexpectedly, the fluorescence spectrum acquired under the same conditions revealed a dramatic shift of the emission maximum from 478 to 554 nm (Figure 1A). The sharp isoemissive point at 549 nm is consistent with a single protonation equilibrium involving the fully deprotonated species and the corresponding conjugate acid. The bathochromic emission shift points toward an ESPT pathway, in which a proton from the isonicotinic acid nitrogen is transferred to the pyridine acceptor of the fluorophore core (Scheme 1). Concluding from a computational analysis of protonated tris(picolyl)amine (Scheme S1, Supporting Information), conformation II containing an intramolecular hydrogen-bond is ∼30 kJ/mol more stable compared to the open conformation I (Tables S1 and S2, Supporting Information). Thus, ESPT might be facilitated through an intramolecular pathway.

**Figure 2:**
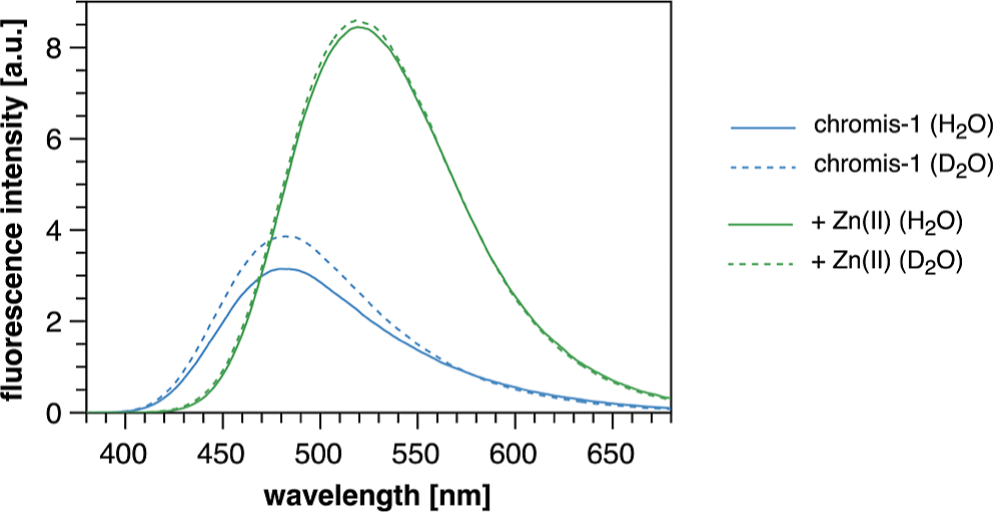
Chromis-1 acid in deuterated vs nondeuterated buffer (10 mM PIPES, 0.1 M KCl, pH/D 7.0, at 25 °C) reveals a solvent deuterium isotope effect. The solid lines show the fluorescence spectra in non-deuterated buffer, whereas the dashed lines represent the fluorescence spectra in deuterated buffer.

To investigate the extent of ESPT at neutral pH, we compared the shape and intensity of the chromis-1 acid emission profile in deuterated vs nondeuterated buffer (10 mM PIPES, 0.1 M KCl, pH/D 7.0, 25 °C). As shown in Figure 2, the emission band in D_2_O was slightly narrower and the intensity increased by 16% compared to H_2_O (blue traces). In contrast, the emission spectrum of Zn(II)-saturated chromis-1 acid remained unaffected when changing the solvent from non-deuterated to deuterated buffer (green traces). Thus, the observed emission increase of free chromis-1 acid in D_2_O indicates that even under neutral conditions, a small but not insignificant fraction of the excited fluorophore engages in ESPT, resulting in a slight broadening of the emission band.

### Intrinsic ESPT

**Scheme 2:**
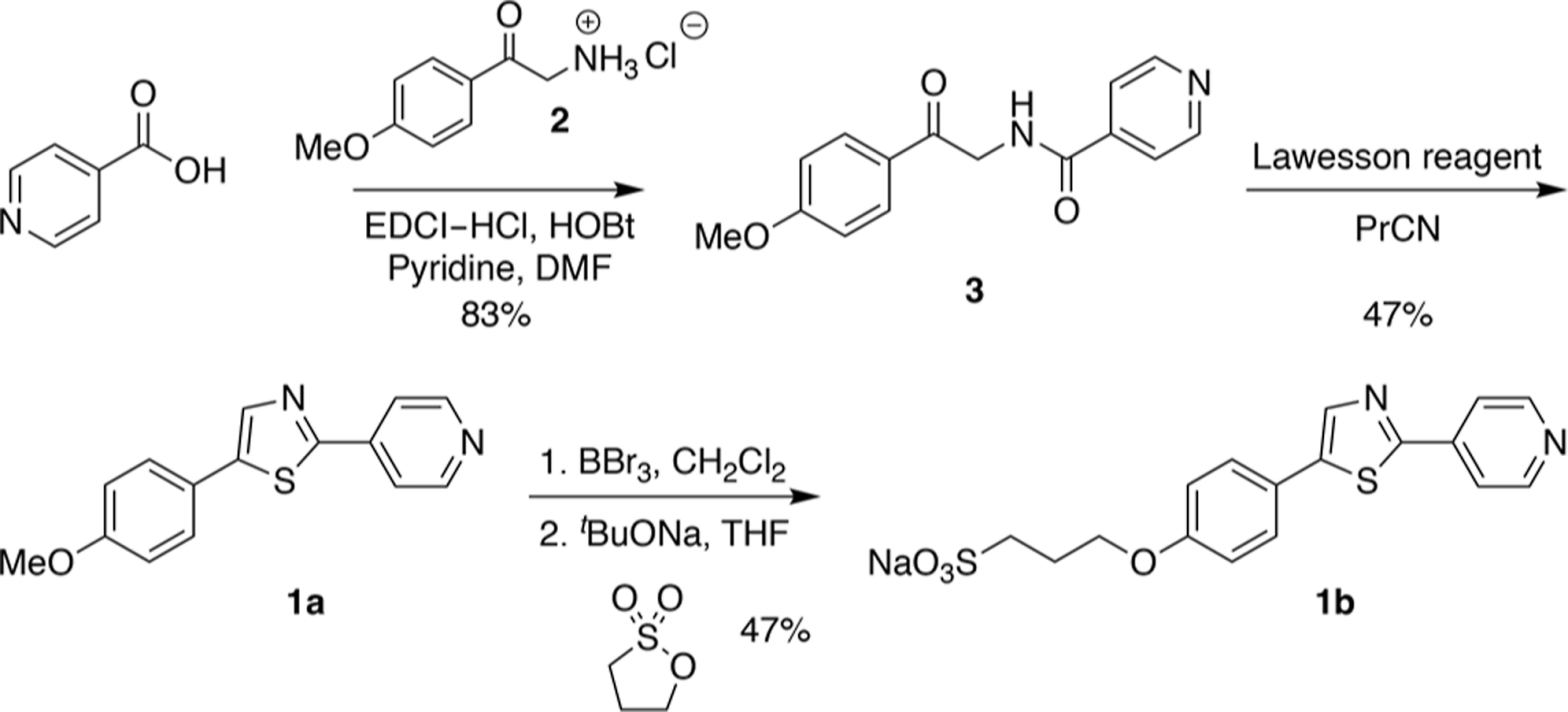


The spectroscopic and computational studies of chromis-1 suggest that the proximity of the protonated bis-isonicotinic acid moiety enables excited-state protonation of the chromophore. To evaluate the intrinsic excited-state basicity of the chromophore, we synthesized a model compound (**1a**, Scheme 2) featuring the fluorophore core of chromis-1 acid but without the chelating moiety. For studies in aqueous buffer, we also synthesized fluorophore **1b** containing a hydrophilic sulfonate group, which increases the water-solubility while also minimizing intermolecular interactions (Scheme 2). To mitigate inductive effects, the sulfonate group was attached to the anisole moiety with a propylene linker.

### Model Compound Synthesis

Starting from commercially available isonicotinic acid, model compound **1a** was accessed in two steps with an overall yield of 33% by coupling with the alpha-amino ketone **2** followed by cyclization with Lawesson’s reagent in butyronitrile (Scheme 2). For the synthesis of **1b**, the methoxy group of **1a** was cleaved with BBr_3_, and the resulting phenol group was alkylated with 1,3-propane sultone. The corresponding sodium salt was isolated in 47% yield starting from **1a**.

### Ground- and Excited-State Protonation of **1a** in Ethanol and 2,2,2-Trifluoroethanol

**Figure 3:**
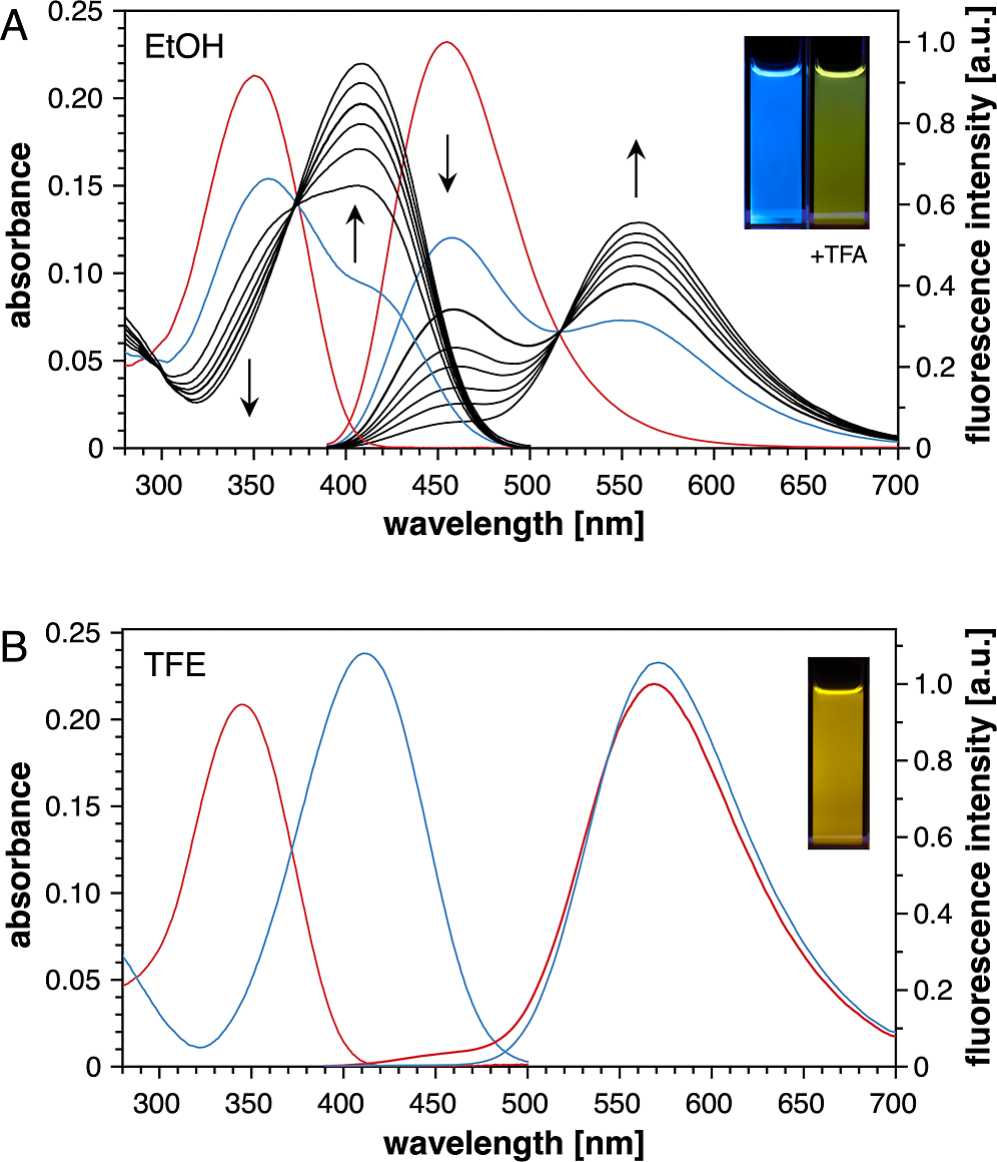
UV–vis absorption (left) and fluorescence emission spectra (right) of model compound **1a** (10 μM) in (A) ethanol and (B) 2,2,2-trifluoroethanol (TFE) under neutral and acidic conditions. The spectra were acquired first in neutral solvent (red traces) and after acidification with 1 mM TFA (blue traces). Inset: Visual appearance of the fluorescence emission before (left) and after (right) acidification. The black traces were recorded at increasing TFA concentrations varying from 2, 5, 10, 20, 50, to 100 mM as indicated by the arrows. Inset: Visual appearance of the fluorescence emission in neutral TFE.

We first evaluated the intrinsic photobasicity of the fluorophore **1a** in ethanol (EtOH), a solvent that is less acidic and less polar compared to water, thereby potentially disfavoring excited-state protonation (Figure 3A). In neutral EtOH, the absorption maximum at 350 nm of **1a** was almost identical compared to chromis-1 at pH 7.0 in aqueous buffer. However, the emission maximum at 455 nm was blue-shifted by 23 nm relative to water, in line with the lower solvent polarity of EtOH. Upon stepwise acidification with trifluoroacetic acid up to a concentration of 100 mM, the absorption maximum shifted from 350 to 410 nm, which is consistent with a stronger ground-state polarization upon protonation of the pyridine nitrogen. The corresponding emission spectra, acquired with excitation at the isosbestic point (374 nm), showed a similarly pronounced shift of the emission maxima from 455 to 559 nm, visibly apparent by a distinct color shift from bright blue to yellow-green (Figure 3A, inset). The sharp isosbestic points in both the absorption and emission spectra indicate a well-defined equilibrium system involving only the neutral and protonated fluorophore. Contrary to the behavior of chromis-1, the gradual emission changes mirror the degree of ground-state protonation upon acidification, indicating negligible ESPT in EtOH.

We next studied the absorption and emission behavior of **1a** in 2,2,2-trifluoroethanol (TFE), which, according to the Kamlet–Taft solvent parameters, is significantly more acidic and less basic than EtOH.^25^ While the absorption maximum of **1a** in neutral TFE was slightly blue-shifted to 445 nm compared to EtOH, the emission spectrum was dominated by a strongly red-shifted band with bright yellow appearance and a maximum at 568 nm (Figure 3B), similar to the spectrum of the protonated fluorophore in acidified EtOH (Figure 3A). Emission from the neutral fluorophore was only apparent as a weak band centered around 450 nm, consistent with almost quantitative ESPT in neutral TFE.

To confirm that the long-wavelength emission band originated from the neutral unprotonated ground-state species, we compared the excitation spectra acquired at 570 and 450 nm (Figure S1, Supporting Information). In agreement with a neutral ground state, the normalized excitation spectra are nearly identical with respect to the shape and position of the absorption maxima. Upon acidification with TFA, the absorption maximum shifted to 411 nm, which is almost identical to the absorption maximum of the protonated fluorophore in ethanol (410 nm). In contrast, the emission spectrum revealed only minor changes upon excitation at the isosbestic point of the absorption traces, thus confirming that the strongly red-shifted emission band under neutral conditions originates from the protonated species. Consistent with the lower basicity of TFE, 1 mM of TFA was already sufficient to achieve quantitative ground-state protonation (Figure 3B, blue trace), whereas in EtOH emission of the unprotonated fluorophore was still evident even at a concentration of 100 mM TFA (Figure 3A). In conclusion, the absorption and fluorescence data indicate that fluorophore **1a** is an effective photobase in TFE but does not promote ESPT in EtOH.

### Ground- and Excited-State Protonation of **1b** in Aqueous Solution

**Figure 4:**
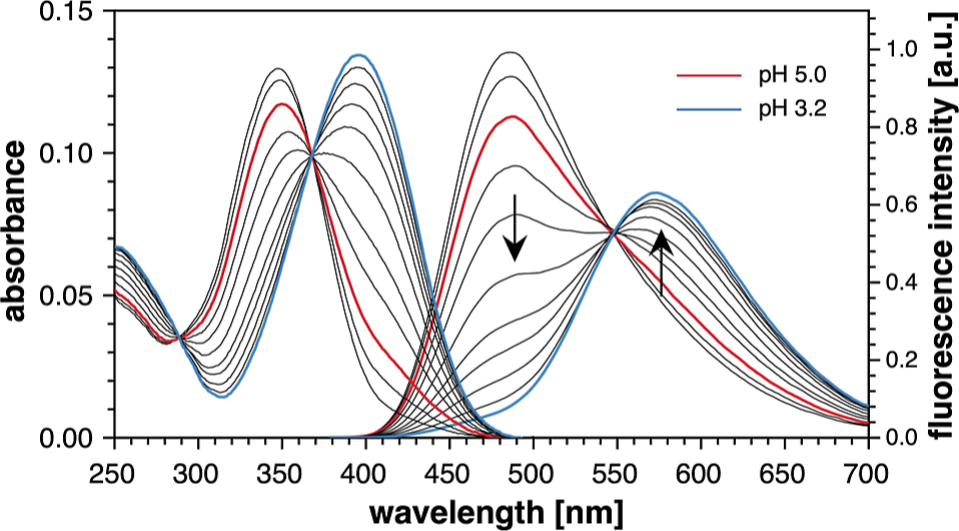
UV–vis absorption (left) and fluorescence emission spectra (right) of model compound **1b** in aqueous solution (0.1 M KCl, 25 °C) with varying pH from 5.6–3.2. The fluorescence spectra were acquired with excitation at the isosbestic point (369 nm).

Upon acidification in aqueous buffer to pH 3.2 (5 mM PIPBS, 0.1 M KCl), the UV–vis spectrum of the water-soluble derivative **1b** underwent a strong bathochromic shift and produced sharp isosbestic points, indicating a well-defined single protonation equilibrium (Figure 4, left traces). The large shift of the absorption maximum from 346 to 397 nm suggests an increase in charge transfer character, which is consistent with ground-state protonation of the pyridine nitrogen. These data also confirm that the first p*K*_a_ of chromis-1 acid (5.5) does not involve protonation of the fluorophore core pyridine nitrogen, since the observed shift is much smaller for chromis-1 compared to **1b**. Nonlinear least-squares fitting of the absorption traces over the entire spectral range yielded a p*K*_a_ of 4.49 ± 0.03 (Figure 4). The lower basicity of **1b** compared to unsubstituted pyridine (p*K*_a_ = 5.2)^26^ is consistent with the electron-withdrawing character of the thiazolyl substituent, which decreases the charge density on the pyridine ring.

In contrast to chromis-1, the fluorescence emission of **1b** underwent only a slight decrease in intensity together with slight broadening of the emission profile upon lowering the pH to 5.0 (Figure 4, red trace). Upon further acidification, both the absorption and emission maxima underwent a significant bathochromic shift, consistent with protonation of the pyridine nitrogen. Importantly, the emission maximum at 573 nm of the protonated model compound **1b** closely matches the fluorescence emission of chromis-1 at pH 5, further supporting excited-state protonation of the pyridine acceptor of chromis-1. Nonlinear least-squares fitting of the emission traces over the entire spectral range yielded a p*K*_a_ of 4.51 ± 0.01, which agrees well with the protonation constant derived from the absorption data. In conclusion, the pH-dependent emission changes reflect the extent of ground-state protonation, and therefore, ESPT in the absence of the bis-isonicotinic acid chelating moiety does not proceed to any significant degree at pH 5.

### Excited-State Protonation of Fluorophore **1b** at Neutral pH

**Figure 5:**
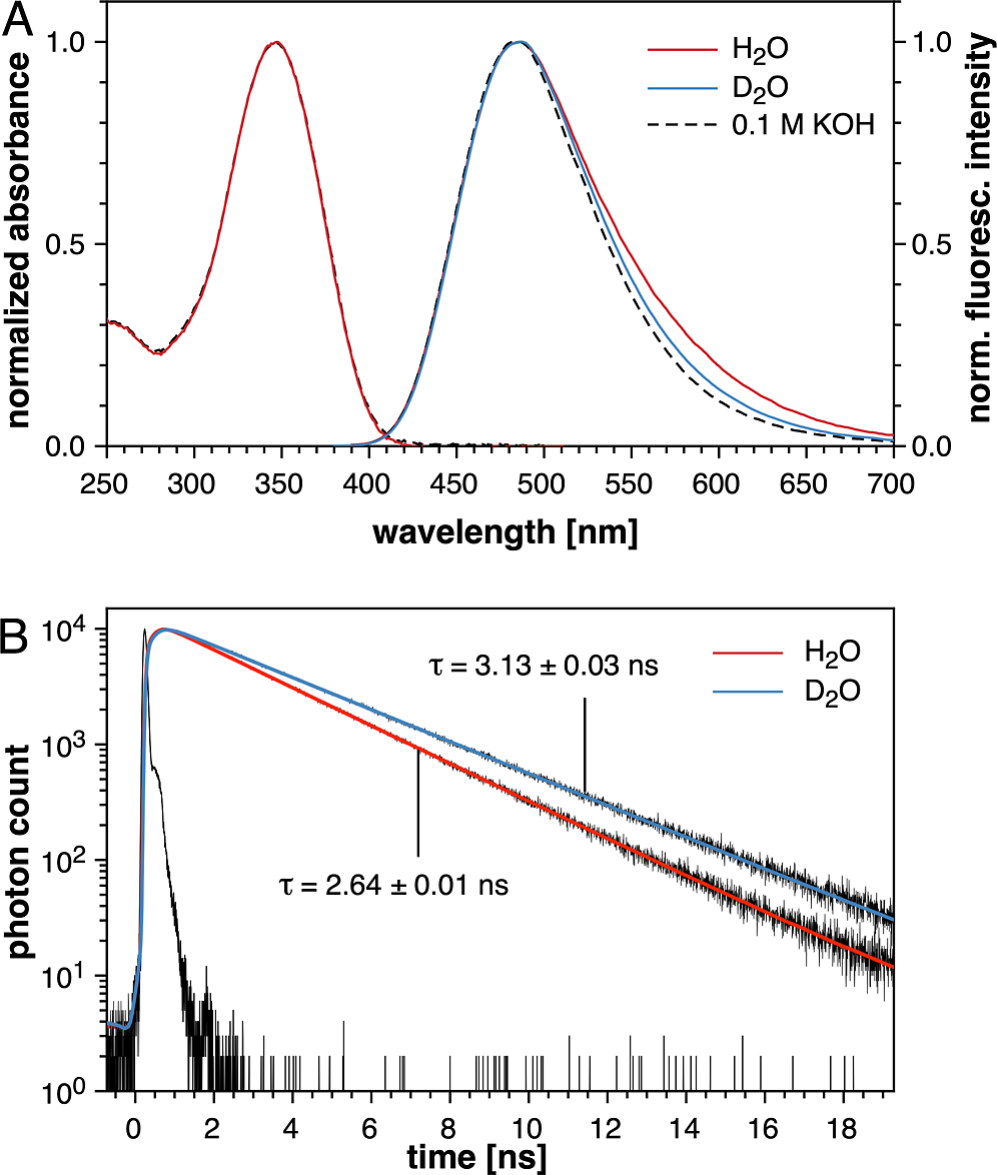
Solvent deuterium isotope effect of **1b** in neutral buffer. (A) Normalized UV–vis absorption (left) and fluorescence emission spectra (right) in aqueous buffer (pH 7.0, 10 mM PIPES, 0.1 M KCl), deuterated buffer (pD 7.0, 10 mM PIPES, 0.1 M KCl), and 0.1 M aqueous KOH at 25 °C. The emission spectra were acquired with excitation at the isosbestic point (369 nm). (B) Time-dependent fluorescence decay profile of **1b** in H_2_O (blue trace) and D_2_O (red trace).

The emission spectrum of **1b** in PIPES buffer at neutral pH revealed an asymmetric band shape with broadening toward the red edge, suggesting a small contribution from the protonated species (Figure 5A, red trace). Under basic conditions in 0.1 M KOH, the broadening decreased, while the shape of the absorption band remained unchanged (Figure 5A, dashed trace). Moreover, the normalized excitation spectra acquired at the blue and red edge of the emission spectrum were superimposable and closely matched the excitation spectrum in 0.1 M KOH (Figure S2). Together, these data suggest that broadening of the emission band is not due to the presence of a small amount of protonated fluorophore formed as part of the ground-state equilibrium but rather is caused by ESPT. To confirm the presence of ESPT at neutral pH, we further acquired an emission spectrum of **1b** in D_2_O (Figure 5A, blue trace). Consistent with almost complete inhibition of ESPT, the emission profile assumed a narrower shape (Figure 5A, blue trace), which closely resembled the spectrum of **1b** in 0.1 M KOH (Figure 5A, dashed trace).

### Time-Resolved Spectroscopy

To gain insights into the ESPT dynamics under neutral conditions, we next acquired time-resolved fluorescence decay profiles of model compound **1b** in H_2_O and D_2_O (Figure 5B). All pertinent data, including quantum yields and derived deactivation rate constants, are presented in Table 2. In both solvents, we observed a clean monoexponential decay profile; however, the 3.13 ns fluorescence lifetime in D_2_O was significantly longer than the 2.64 ns lifetime in H_2_O. Concomitant with the longer lifetime, the quantum yield also increased from 0.65 in water to 0.80 in D_2_O. Concluding from the derived excited-state deactivation rate constants, the increase in quantum yield is due to a sharp decrease in the non-radiative deactivation rate from 1.3 × 10^8^ s^–1^ (H_2_O) to 6.4 × 10^7^ s^–1^ (D_2_O), whereas the radiative deactivation rate remains essentially unchanged between the two solvents. These data, combined with the similarities between the emission spectra of **1b** in D_2_O and 0.1 M KOH as well as the increased quantum yield (0.8), suggest that ESPT is effectively suppressed in D_2_O. Thus, the difference in the non-radiative rate constants between H_2_O and D_2_O can serve as a lower estimate of the ESPT rate constant, yielding
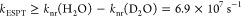


**Table 1:** Photophysical and Thermodynamic Properties of Model Compound **1b** in Aqueous Solution^a^

	**1b**	[**1b**-H^+^]^b^
absorption λ_max_ [nm]^c^	346	397
ε [10^4^ M^–1^ cm^–1^]^d^	2.3	2.5
emission λ_max_ [nm]	489	581
*ṽ*_00_ [cm^–1^]^e^	24,630	21,155
ground state p*K*_a_		4.49 ± 0.03
excited state p*K*_a_*^f^		11.7

a 10 mM PIPES, 0.1 M KCl, 25 °C.

b Fully protonated **1b** in 0.1 M HCl.

c Lowest-energy band of the absorption spectrum.

d Molar extinction coefficient at λ_max_.

e Based on λ^2^-corrected emission spectra.

f Estimated based on eq 2.

**Table 2:** Time-Resolved Fluorescence Decay Data and Excited-State Deactivation Rate Constants for Model Fluorophore **1b** in H_2_O and D_2_O at 22 °C^d^^e^

	H_2_O	D_2_O
τ_F_ [ns]^a^	2.64 ± 0.01	3.13 ± 0.03
goodness of fit (χ^2^)^b^	1.245	1.298
quantum yield (Φ_F_)^c^	0.65	0.80
*k*_r_ [10^8^ s^–1^]^a^	2.46	2.56
*k*_nr_ [10^8^ s^–1^]^b^	1.33	0.64

a Fluorescence lifetime.

b Monoexponential decay model.

c Quinine sulfate as reference.

d Radiative deactivation rate constant *k*_r_ = Φ_F_/τ_F_.

e Non-radiative deactivation rate constant *k*_nr_ = (1 – Φ_F_)/τ_F_.

Altogether, the time-resolved fluorescence data combined with the steady-state fluorescence studies paint a consistent picture in which the model fluorophore **1b** undergoes ESPT under neutral conditions, albeit only to a small degree relative to fluorescence emission from the neutral form.

### Thermodynamics of Excited-State Protonation

To estimate the driving force of the excited-state protonation of fluorophore **1b**, we employed a quasi-thermodynamic Förster cycle,^27,28^ which relates the excited state p*K*_a_* with the ground-state protonation equilibrium constant through the electronic (0,0) transition energies *ṽ*_00_ of the neutral (B) and protonated forms (BH) of the fluorophore according to eq 1
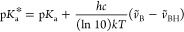
1wherein *k* is the Boltzmann constant, *T* the temperature, *c* the speed of light, and *h* is the Planck’s constant. This expression assumes that the entropy change of the protonation reaction is similar in the ground and excited states, which is a valid approximation in most cases.^28^ At *T* = 298 K, eq 1 can be simplified to
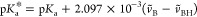
2with *ṽ*_B_ and *ṽ*_BH_ expressed in units of wavenumbers (cm^–1^). The electronic transition energies *ṽ*_00_ of the neutral and protonated fluorophore were approximated as the average of the peak absorption and emission energies. To determine the emission maxima in units of wavenumbers, the fluorescence spectra were corrected by multiplying the wavelength-depen-dent emission intensity with λ^2^.^29^ Based on the data compiled in Table 1, we estimated an excited state p*K*_a_* of 11.7, which is 7 orders of magnitude higher than the ground state p*K*_a_. Thus, the p*K*_a_ increase upon photoexcitation of fluorophore **1b** compares well with established photobases such as quinoline, which undergoes a similar p*K*_a_ increase from 4.8 to 11.2 upon photoexcitation.^30^

### Solvatochromic Shift and Excited-State Dipole Moment

The photobasicity of **1b** implies a dramatic redistribution of the π-electron density upon transition from the ground to the excited state. To estimate the degree of excited-state polarization, we performed a solvatochromic shift analysis. Accordingly, we acquired UV–vis absorption and fluorescence emission spectra of the charge-neutral model compound **1a** in a series of solvents covering a large dielectric range from 2.0 to 35.9. The spectral data were then analyzed based on the Lippert–Mataga formalism,^31−33^ which relates the experimental Stokes shift *ṽ*_abs_ – *ṽ*_em_ with the solvent polarity function Δ*f* = *f*(ϵ_*r*_) – *f*(*n*) and the difference between the excited-state and ground-state dipole moments (μ_e_ – μ_g_) according to eq 3
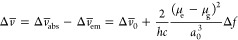
3with
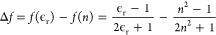
4where ϵ_*r*_ and *n* are the relative permittivity and refractive index, respectively, and Δ*ṽ*~_0_ corresponds to the extrapolated Stokes shift in vacuum.

**Figure 6:**
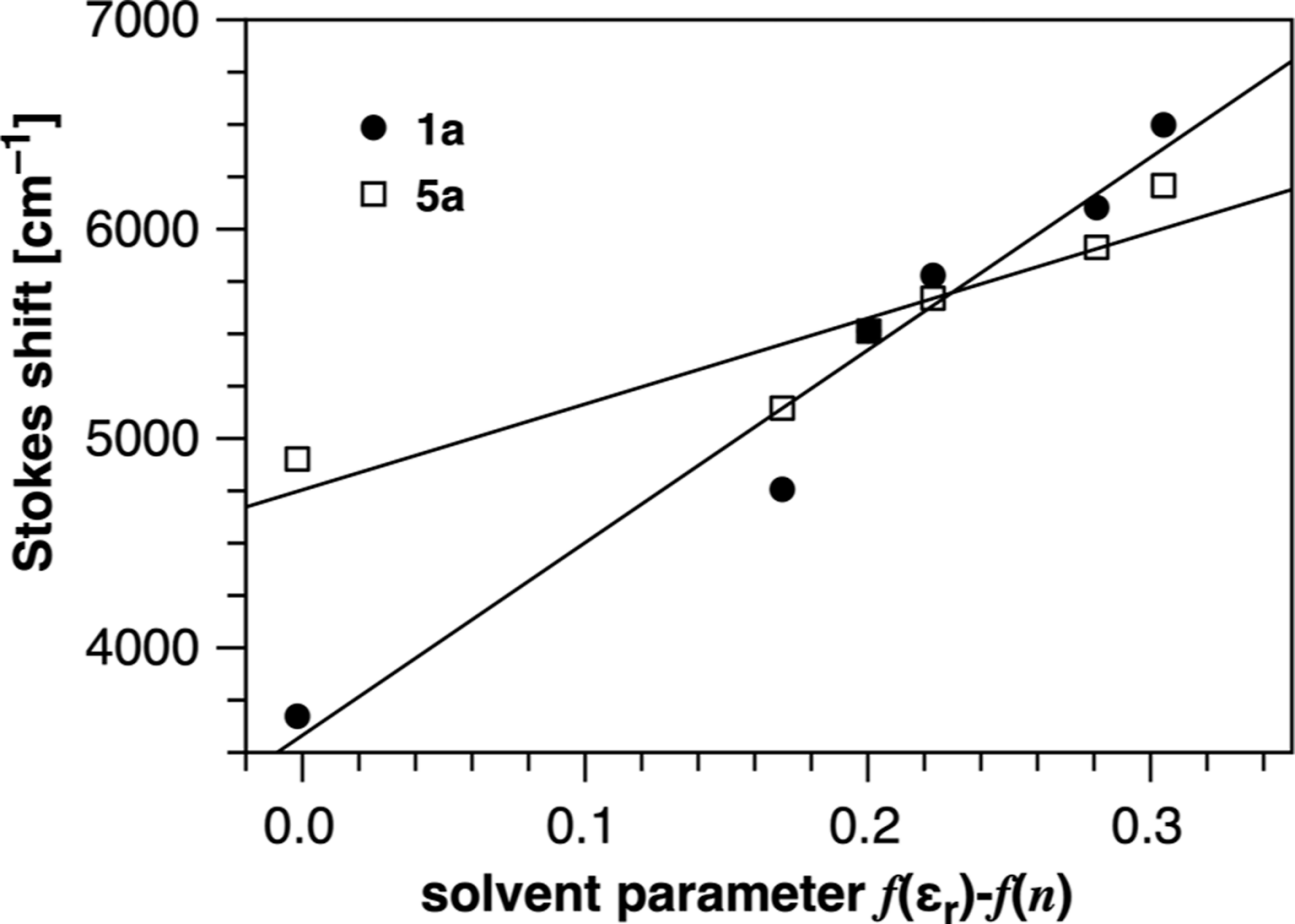
Solvent-dependent Stokes shift as a function of solvent polarity of model fluorophore **1a** (filled circles) and **5a** (squares). The data were analyzed based on the Lippert-Mataga formalism according to eq 3 to determine the increase in dipole moment (μ_e_ – μ_g_) upon photoexcitation.

As described above for the Förster cycle, the solvent-dependent emission spectra were converted to a wavenumber scale by applying the λ^2^ correction.^29^ The Onsager cavity radius a_0_ = 5.04 Å was estimated based on the computational molar volume. Plotting Δν̅ vs Δ*f* followed by a linear regression analysis yielded a slope of 9200 ± 930 cm^–1^ (*r* = 0.980), based on which we determined an increase in the dipole moment upon photoexcitation (μ_e_ – μ_g_) of 10.8 ± 0.5 D (Figure 6). Thus, the significant excited-state polarization is commensurate with the observed photobasicity of the chromis-1 fluorophore core.

### Computational Studies

**Figure 7:**
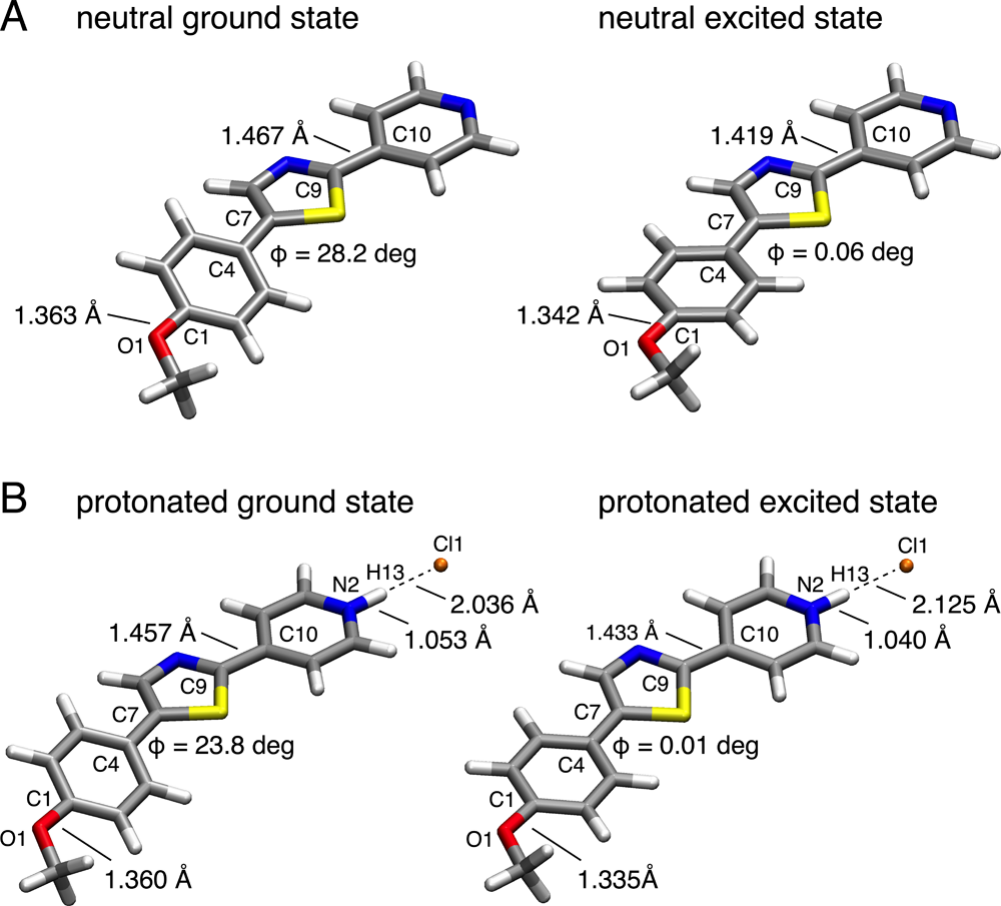
Energy-minimized structures of the neutral (A) and protonated (B) fluorophore **1a** in the ground and excited state based on quantum chemical calculations at the B3LYP/6-31+G(d) level of theory and polarized continuum model (PCM) to account for solvent effects.

To gain further insights into the extent of ground- and excited-state polarization of the chromis-1 fluorophore core, we performed a series of DFT quantum chemical calculations. To this end, we energy-minimized the geometry of fluorophore **1a** at the B3LYP/6-31+G(d) level of theory and estimated the vertical excitation energy based on a TD-DFT single-point calculation using the long-range corrected CAM-B3LYP functional and the 6-31+G(d) basis set with added diffuse functions.^19,34^ Moreover, we estimated the fluorescence emission energy at the same level of theory based on the TD-DFT energy-minimized geometry of the lowest excited state. As illustrated in Figure 7, the fluorophore **1a** assumes in the ground state a nonplanar geometry with a dihedral angle of 28° (Figure 7A), thus mirroring the twisted geometry of chromis-1 observed in the X-ray crystal structure.^12^ In contrast, the lowest-energy excited state (S1) assumes a planar geometry, which is likely a result of electron delocalization from the anisole donor moiety toward the pyridine ring. The increased excited-state polarization is also evident from the shortening of the O1–C1, C4–C7, and C9–C10 bonds (Table 3) and the increase in the permanent dipole moment from 5.5 to 11.7 D. The estimated gas phase energies of the vertical excitation and fluorescence emission were both overestimated by an average of 0.34 eV compared to the experimental values.

**Table 3:** Computational Data for Fluorophore **1a** in the Gas Phase and with PCM Solvent Equilibration in Water

parameter	gas phase^a^		water^a^^,^^b^	
	GS	S1^c^	GS	S1^c^
C9–C10 [Å]^d^	1.467	1.427	1.468	1.419
C4–C7 [Å]^d^	1.466	1.428	1.466	1.421
O1–C1 [Å]^d^	1.363	1.351	1.363	1.342
C3–C4–C7–C8 [deg]^e^	–28.2	0.06	–24.9	–0.02
μ [D]^f^	5.5	11.7	7.1	17.5
*E* [eV] (TD-DFT)^g^	3.86	3.20	3.69	2.78
Osc. strength	0.826	0.875	0.935	1.195
*E* [eV] (exp)	n.d	n.d	3.58	2.53^h^

a Geometry optimized at B3LYP/6-31+G(d) level of theory.

b PCM solvent equilibration.

c Excited-state geometry optimized via TD-DFT (B3LYP/6-31+G(d)) with and without PCM solvent equilibration.

d Bond length.

e Dihedral angle.

f Permanent dipole moment.

g Vertical transition energy (CAM-B3LYP/6-31+G(d)//B3LYP/6-31+G(d)).

h λ^2^-Corrected emission maximum.

**Figure 8:**
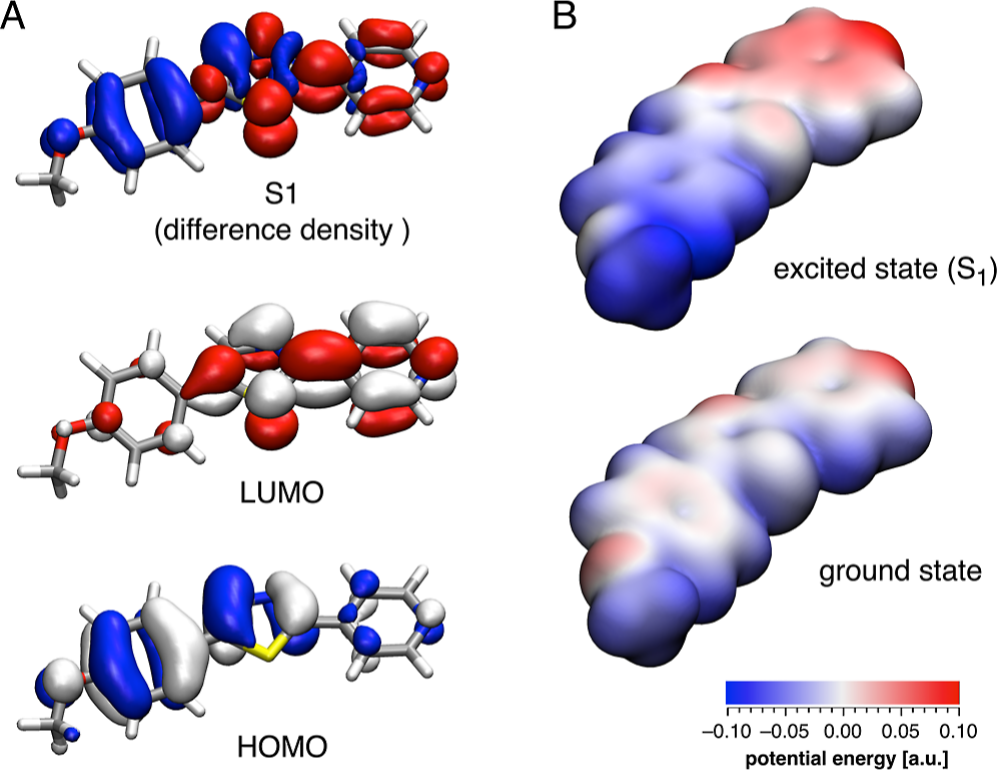
(A) Frontier orbitals and a plot of the electron density difference between ground and excited state of fluorophore **1a** calculated based on TD-DFT at the CAM-B3LYP/6-31+G(d)//B3LYP/6-31+G(d) level of theory including PCM solvent equilibration. (B) Ground- and excited-state electrostatic potential distribution mapped on an iso-electron density surface of fluorophore **1a**.

To account for solvent polarization effects, we applied a polarized continuum model (PCM),^20^ which reproduced the experimental data with an improved mean average error of 0.18 eV (Table 3). Moreover, upon PCM solvent equilibration, the permanent dipole moment increased from 5.5 to 7.1 D in the ground state and from 11.7 to 17.5 D in the excited state. Altogether, the PCM-corrected model estimated an increase in the dipole moment by 10.4 D upon photoexcitation, which is in excellent agreement with the experimental value of 10.7 ± 0.5 D from the Lippert-Mataga analysis. A plot of the HOMO and LUMO densities further revealed that the excited-state polarization is a direct consequence of their spatial localization (Figure 8A). Likewise, the total electron density difference between the ground and lowest excited state mirrors the difference between the frontier orbital densities, thus demonstrating that the vertical excitation is dominated by a HOMO–LUMO electronic transition. We further visualized the increase of the polarization of **1a** upon excitation by an electrostatic potential map superimposed on an iso-electron density surface (Figure 8B), which revealed large shift of the charge density from the anisole moiety toward the pyridine nitrogen.

To evaluate the effect of protonation on the ground and excited state properties, we performed analogous calculations with PCM solvent equilibration using the hydrochloride salt **1a**·HCl as the model compound (Table 4). Consistent with an increase in ground state polarization, the bond lengths O1–C1, C4–C7, and C9–C10, as well as the dihedral angle C3–C4–C7–C8, decreased compared to neutral **1a** (Figure 7B). Moreover, the marked decrease of the N2–H13 bond length from 1.053 to 1.040 Å upon excitation further substantiates the photobasicity of the pyridine nitrogen. Concomitant with shorting of the N–H bond, the H-bonding interaction with the chloride anion decreased in the excited state as indicated by the increase in bond lengths from 2.036 (GS) to 2.125 Å (S1). Similar to neutral **1a**, a TD-DFT calculation at the CAM-B3LYP/6-31+G(d) level of theory reproduced the vertical excitation energy within 0.14 eV; however, the emission energy was overestimated by almost 0.7 eV compared to the experimental data (Table 4). In contrast, the vertical transition energy with B3LYP performed significantly better with an error of only 0.13 eV.

**Table 4:** Computational Data for Protonated Fluorophore **1a**·HCl with PCM Solvent Equilibration

parameter	GS^a^	S1^b^
C9–C10 [Å]^c^	1.457	1.433
C4–C7 [Å]^c^	1.462	1.434
O1–C1 [Å]^c^	1.360	1.335
N2–H13 [Å]^c^	1.053	1.040
H13 Cl1 [Å]^c^	2.036	2.125
C3–C4–C7–C8 [deg]^d^	23.8	0.01
μ [D]^e^	22.6	25.7
*E* [eV] (TD-DFT)^f^	3.26	2.80 (2.26)^g^
Osc. strength	0.974	0.684 (1.143)^g^
*E* [eV] (exp)	3.12	2.13^h^

a Geometry optimized at B3LYP/6-31+G(d) level of theory.

b Excited-state geometry optimized via TD-DFT (B3LYP/6-31+G(d)).

c Bond length.

d Dihedral angle.

e Permanent dipole moment.

f Vertical transition energy based on CAM-B3LYP/6-31+G(d)//B3LYP/6-31+G(d).

g Vertical transition energy and oscillator strength based on (B3LYP/6-31+G(d)//B3LYP/6-31+G(d)).

h λ^2^-Corrected emission maximum.

### Redesigning the Chromis-1 Fluorophore Core

**Chart 2:**
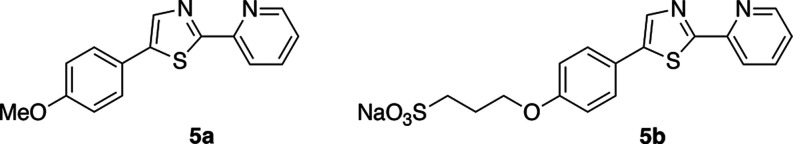


The computational studies of the model fluorophore **1a** revealed a remarkable excited-state polarization, which is likely responsible for promoting ESPT in chromis-1. Thus, we reasoned that the pH-dependence of the chromis-1 emission might be mitigated by reducing the electron-withdrawing character of the pyridine moiety. To this end, we moved the attachment point of the π-bridging thiazole from the para- to the ortho-position of the pyridine acceptor (Chart 2). We expected that this minimal structural change would attenuate the degree of charge delocalization and lower both the ground- and excited-state basicity.^35^ Moreover, positioning of the thiazole nitrogen adjacent to the pyridine nitrogen might provide an additional nitrogen donor for Zn(II)-coordination, thus potentially enhancing the binding affinity compared to chromis-1.

Given the close agreement between the computational and experimental data of the model compounds **1a** and **1b**, we again estimated the degree of excited-state polarization of **5a** based on DFT calculations. Analogous to the previous results, the excited-state polarization was more pronounced in a polar solvent environment compared to the gas phase (Table 5). Most importantly, the ground-state dipole moment of **5a** was only 1.8 D, a 75% reduction over the para-substituted derivative. At the same time, there was still a significant degree of charge transfer toward the pyridine moiety in the excited state (S1), as indicated by the increased dipole moment of 9.6 D (Table 5).

**Table 5:** Computational Data for Fluorophore **5a** in the Gas Phase and with PCM Solvent Equilibration in Water

parameter	gas phase^a^		water^a^^,^^b^	
	GS	S1^c^	GS	S1^c^
μ [D]^d^	1.4	6.7	1.8	9.6
*E* [eV] (TD-DFT)^e^	3.85	3.19	3.72	2.78
Osc. strength	0.850	0.882	0.951	1.208

a Geometry optimized at B3LYP/6-31+G(d) level of theory.

b PCM solvent equilibration.

c Excited-state geometry optimized via TD-DFT (B3LYP/6-31+G(d)) with and without PCM solvent equilibration.

d Permanent dipole moment.

e Vertical transition energy (CAM-B3LYP/6-31+G(d)//B3LYP/6-31+G(d)).

Encouraged by the computational results, we then characterized the photophysical properties of the chromophore **5a** and the water-soluble analog **5b** (Table 6), which were synthesized from picolinic acid analogous to model compounds **1a** and **1b** (Scheme S2, Supporting Information). Solvatochromic shift analysis of **5a** in organic solvents of increasing polarity revealed a slope of 4100 ± 830 cm^–1^ (*r* = 0.927), which, according to the Lippert–Mataga formalism, resulted in a dipole moment increase (μ_e_ – μ_g_) of 7.3 ± 0.7 D upon excitation (Onsager cavity radius *a*_0_ = 5.07 Å). These data also agreed well with the dipole moment increase of 7.8 D obtained from DFT calculations. At neutral pH, the absorption maximum of fluorophore **5b** is with 345 nm essentially identical compared to **1b**.

**Table 6:** Photophysical and Thermodynamic Properties of Model Compound **5b** in Aqueous Solution^a^

	**5b**	[**5b**-H^+^]^b^
absorption λ_max_ [nm]^c^	345	386
ε [10^4^ M^–1^ cm^–1^]^d^	2.3	2.1
emission λ_max_ [nm]	467	565
*ṽ*_00_ [cm^–1^]^e^	21,100	17,360
ground state p*K*_a_		2.34 ± 0.03
excited state p*K*_a_*^f^		9.4

a 10 mM PIPES, 0.1 M KCl, 25 °C.

b Fully protonated **5b** in 0.1 M HCl.

c Lowest-energy band of the absorption spectrum.

d Molar extinction coefficient at λ_max_ based on λ^2^-corrected emission spectra.

f Estimated based on eq 2.

**Figure 9:**
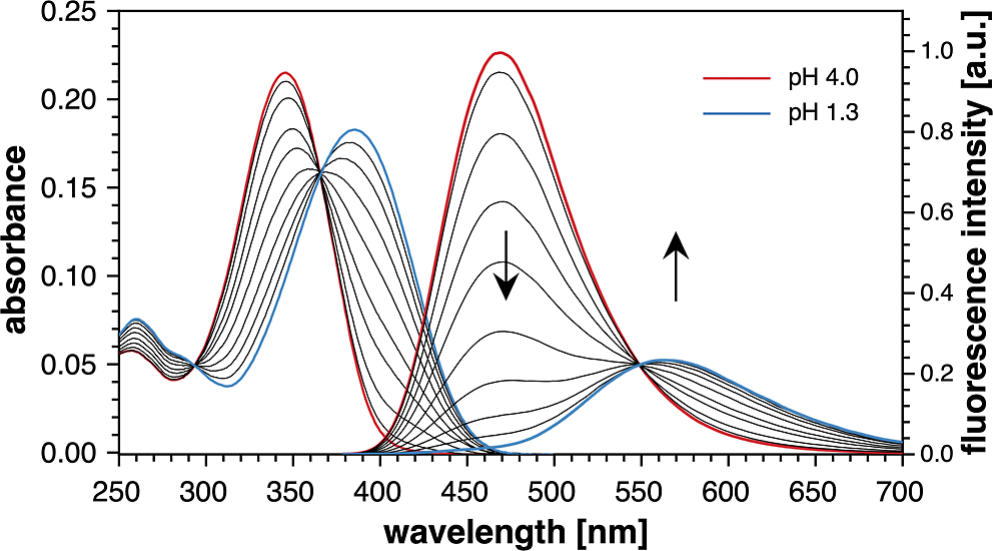
UV–vis absorption (left) and fluorescence emission spectra (right) of model compound **5b** in aqueous solution (0.1 M KCl, 25 °C) as a function of pH varying from 4.0–1.3. The fluorescence spectra were acquired with excitation at the isosbestic point (369 nm).

Consistent with a reduced excited-state polarization, the emission maximum of **5b** was blue-shifted by 22 nm from 489 nm (**1b**) to 467 nm (**5b**). Upon acidification, the peak absorption and emission of **5b** responded with comparable spectral shifts of 41 and 98 nm, respectively (Figure 9), although both maxima were blue-shifted relative to **1b**. Nonlinear least-squares fitting of the pH-dependent absorption traces yielded a p*K*_a_ of 2.34 ± 0.03, which is more than two orders of magnitude lower compared to **1b**. Employing a quasi-thermodynamic Förster cycle, we further estimated an excited-state p*K*_a_* of 9.4, which corresponds to a reduction in basicity by nearly two orders of magnitude compared to **1b**.

**Figure 10:**
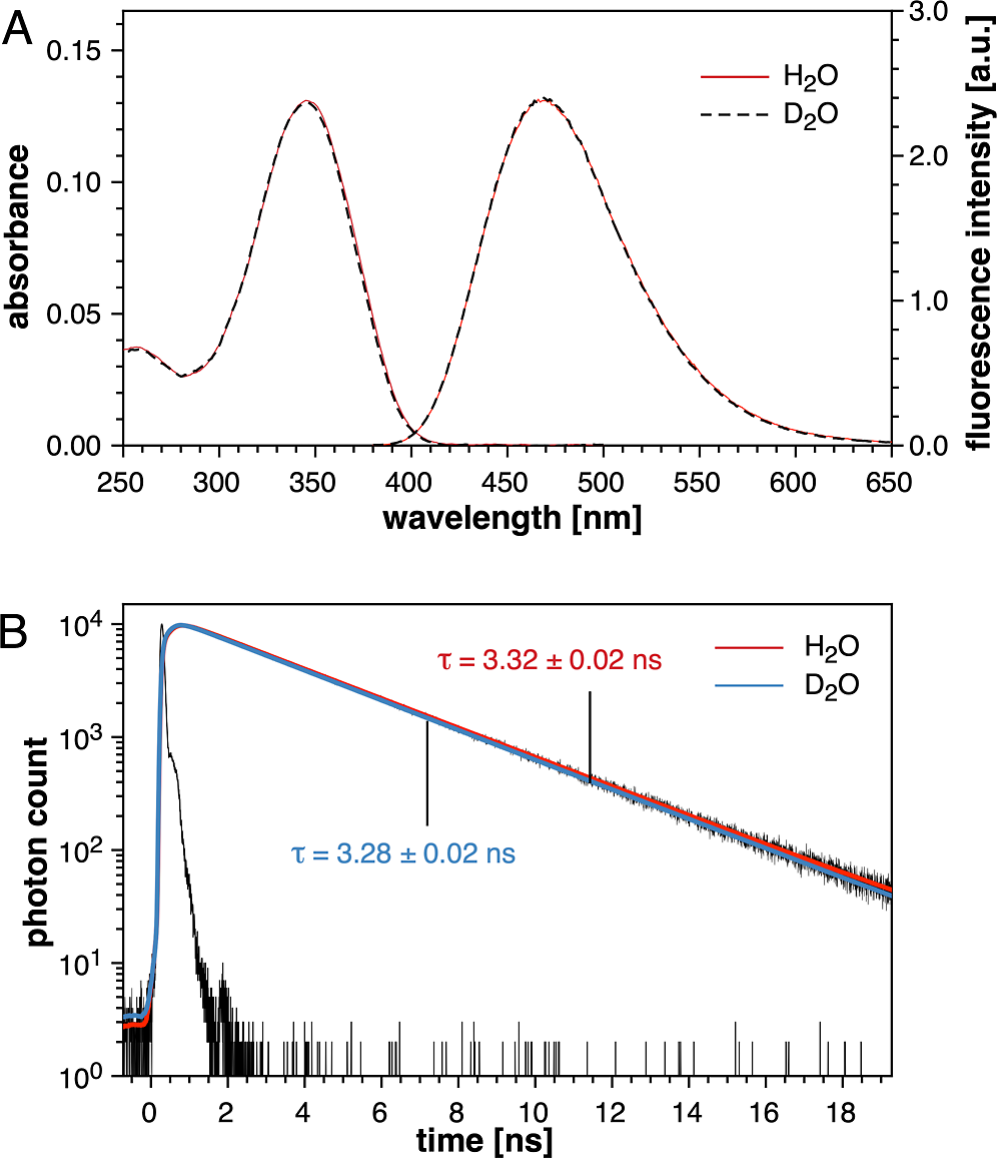
Solvent deuterium isotope effect of **5b** in neutral aqueous buffer. (A) Normalized UV–vis absorption (left) and fluorescence emission spectra (right) in aqueous buffer (pH 7.0, 10 mM PIPES, 0.1 M KCl) and deuterated buffer (pD 7.0, 10 mM PIPES, 0.1 M KCl) at 25 °C. The emission spectra were acquired with excitation at the isosbestic point (369 nm). (B) Time-dependent fluorescence decay profile of **5b** in H_2_O (red trace) and D_2_O (blue trace).

Finally, we explored the solvent deuterium isotope effect of model fluorophore **5b** by steady-state and time-resolved fluorescence spectroscopy (Figure 10). In contrast to fluorophore **1b**, the emission spectra of **5b** in D_2_O did not change in either intensity or shape compared to H_2_O. Moreover, the fluorescence lifetime of **5b** was, within experimental error, identical in both solvents. Notably, the fluorescence lifetime of 3.3 ns for **5b** is significantly longer compared to **1b**, which in turn is consistent with the increase in quantum yield from 0.65 for **1b** to 0.8 for **5b**. Altogether, the absence of a solvent deuterium isotope effect is consistent with the reduced excited-state basicity of the redesigned fluorophore core, indicating effective suppression of ESPT in aqueous solution at neutral pH.

### Relative Fluorophore Brightness in TPEM

**Figure 11:**
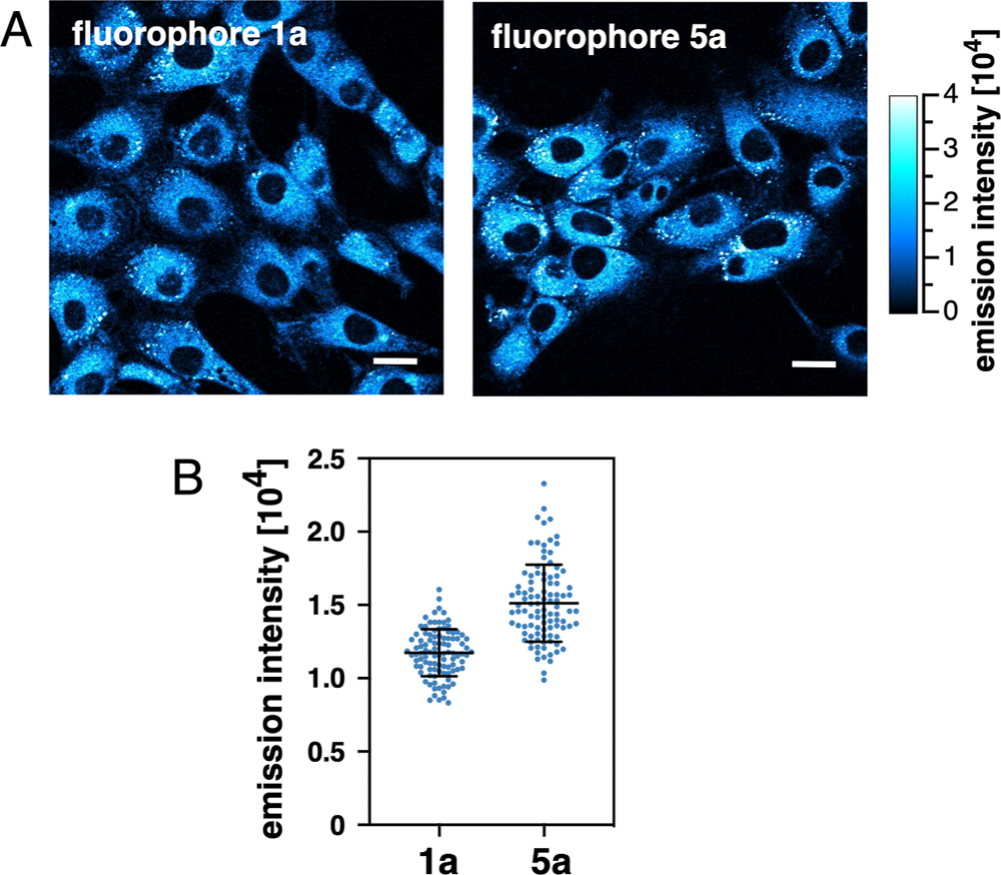
Relative brightness of fluorophores **1a** and **5a** in TPEM. (A) Two-photon-excited fluorescence micrographs of 3T3 mouse fibroblasts after incubation with 2 μM fluorophore **1a** (left) or **5a** (right) for 30 min (λ_ex_ = 720 nm, 1% laser power, emission collected from 425–704 nm with a 40×/1.30 oil objective). Scale bar: 20 μm. (B) Emission intensity distribution of 100 individual cells. The black bars indicate the mean ± standard deviation. The intensity scale is based on a 16 bit image resolution with a maximum value of 65,535.

To evaluate the relative brightness of the redesigned fluorophore **5b** compared to **1b**, we incubated 3T3 mouse fibroblasts with each fluorophore at a concentration of 2 μM for 30 min (37 °C) and acquired two-photon excited fluorescence micrographs using a Zeiss LSM 710 confocal NLO microscope. As illustrated with Figure 11, both fluorophores readily entered cells and produced a bright intracellular emission upon two-photon excitation at 720 nm. To compare the relative brightness of the two fluorophores, we collected the fluorescence emission over the entire spectral range from 425–704 nm for 100 individual cells. Based on the 16 bit detector resolution, we measured an average emission intensity for cells incubated with **5a** of 15,100 ± 2,600, which corresponds to an approximate 28% increase over **1a** with an average of 11,700 ± 1600. While we cannot assume that both fluorophores are internalized to yield the same intracellular concentrations, the similar brightness still indicates that the redesigned fluorophore architecture of **5a** does not significantly compromise the two-photon sensitivity in cellular imaging.

## Conclusions

The comprehensive photophysical characterization and computational studies described herein revealed that the emission-shift of chromis-1 upon acidification is due to an ESIPT pathway assisted by the pendant bis-isonicotinic acid moiety. The observed solvatochromic shift is consistent with a large increase in the dipole moment upon photoexcitation, resulting in a polarized charge-transfer state with increased charge density at the pyridine nitrogen. Although the p*K*_a_ of the pyridine acceptor in the excited state is more than six orders of magnitude higher than in the ground state, its photobasicity is not sufficient to promote an effective intermolecular ESPT from solvent water molecules at neutral pH. The ESIPT pathway of chromis-1 resembles the behavior of the recently described “strapped” ESIPT fluorophore system, in which a pyridine photobase with donor–accep-tor–donor architecture was tethered to a basic tertiary trialkylamine.^36^ Analogous to chromis-1, the tertiary amine rather than the pyridine moiety is protonated upon acidification to form an intramolecular hydrogen bond, which then facilitates ESIPT upon photoexcitation. By attaching the thiazole π-bridge in the ortho-rather than the para-position of the pyridine acceptor, the photobasicity was reduced by two orders of magnitude while maintaining the two-photon-excited brightness for cellular imaging applications. The results of this study underscore the importance of the pendant chelating moiety in minimizing potential pH-dependent responses of two-photon probes and may offer valuable guidelines for the design of metal-ion selective probes for biological imaging.

## Supporting Information Available

NMR spectra, solvent-dependent absorption and emission spectra, and computational data (PDF)

## Abbreviations

TPEM: two-photon excitation microscopy
TD-DFT: time-dependent density functional theory
PCM: polarized continuum model
ESPT: excited-state proton transfer
ESIPT: excited-state intramolecular proton transfer
