# Supplementary material for: Balancing Brightness and Photobasicity: Modulating Excited-State Proton Transfer Pathways in Push–Pull Fluorophores for Biological Two-Photon Imaging
Source: J Phys Chem A. 2024 Nov 7;128(46):9904–16. doi: 10.1021/acs.jpca.4c05649 (PMC11586903; doi:10.1021/acs.jpca.4c05649)
Supplement: Supplementary file 1 — jp4c05649_si_001.pdf [file jp4c05649_si_001.pdf]

Supplementary Information for

Balancing Brightness and Photobasicity: Modulating Excited-State Proton Transfer Pathways in Push-Pull Fluorophores for Biological Two-Photon Imaging

Adam M. McCallum, Jiyao Yu, S. Sumalekshmy, Abigail Hagwood, and Christoph J. Fahrni*

School of Chemistry and Biochemistry and Petit Institute for Bioengineering and Bioscience, Georgia Institute of Technology, 901 Atlantic Drive, Atlanta, Georgia 30332

Table of Contents

1. ^{1}H-NMR and ^{13}C-NMR spectra S2

2. Supplementary Schemes S1 and S2 S10

3. Supplementary Figures S1-S4 S11

4. Supplementary Tables S1-S14 S13

I. NMR Spectra

^{1}H NMR ($CDCl_3$, 400 MHz)

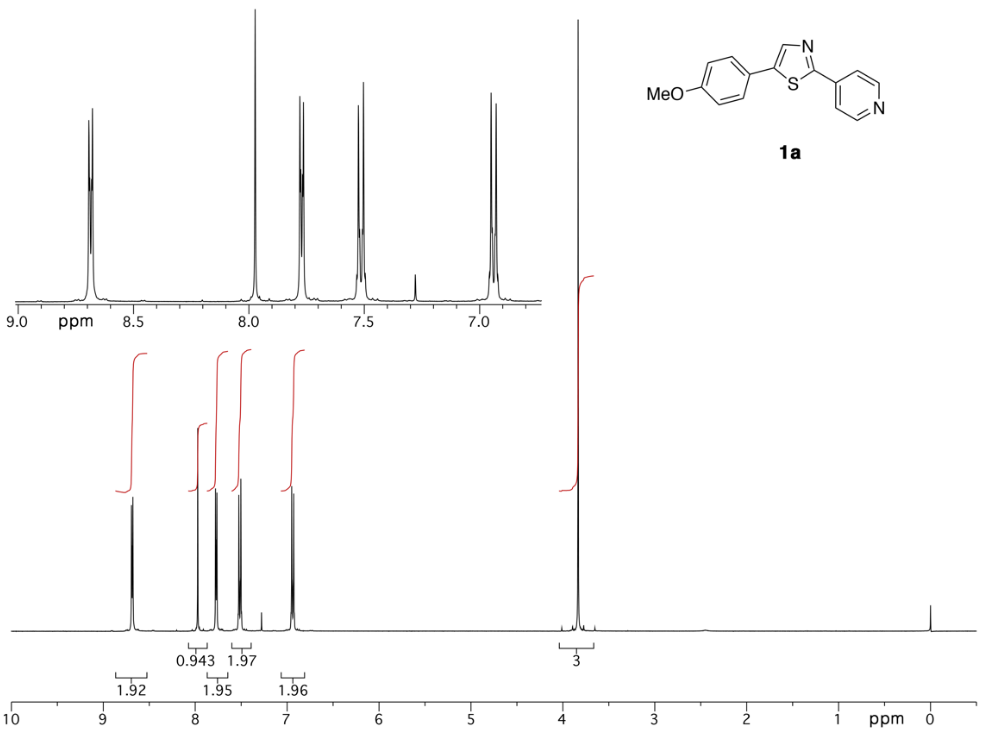

^{13}C NMR ($CDCl_3$, 100 MHz)

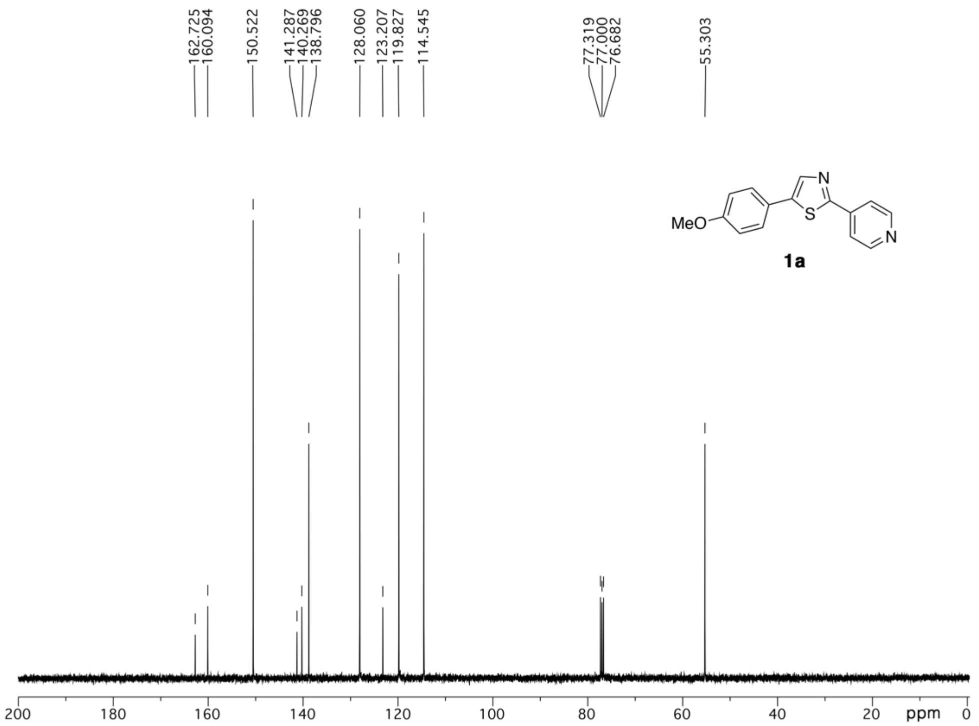

^{1}H NMR (DMSO-d$_6$, 400 MHz)

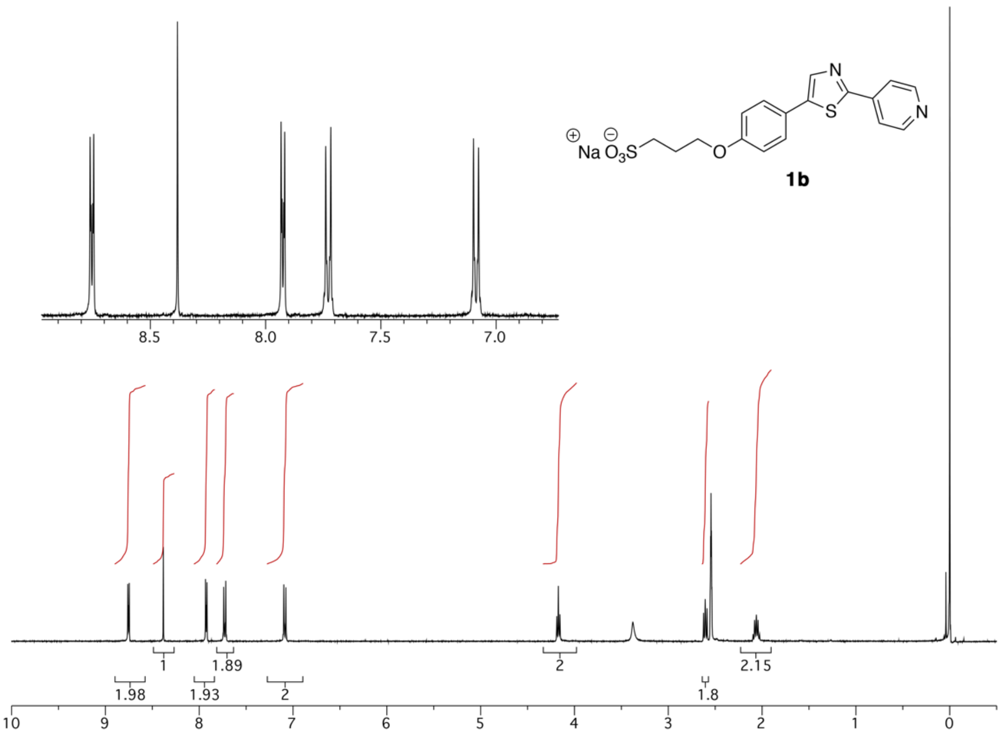

^{13}C NMR (DMSO-d$_6$, 100 MHz)

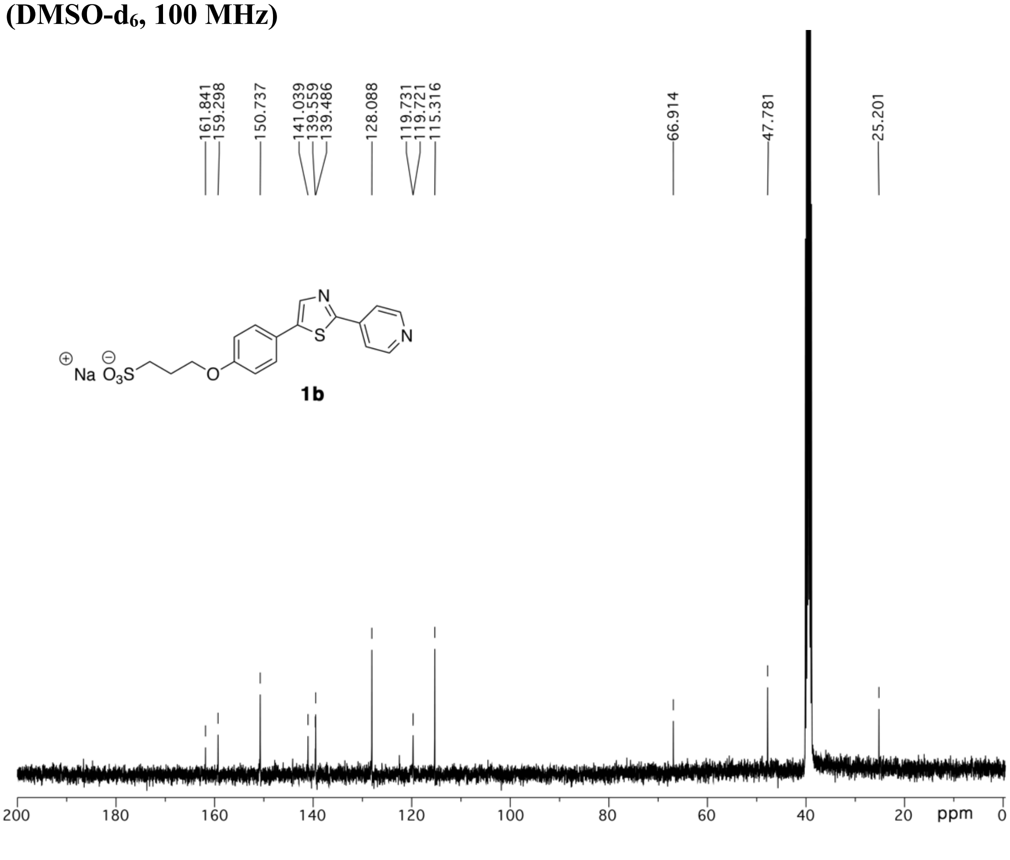

^{1}H NMR ($CDCl_3$, 400 MHz)

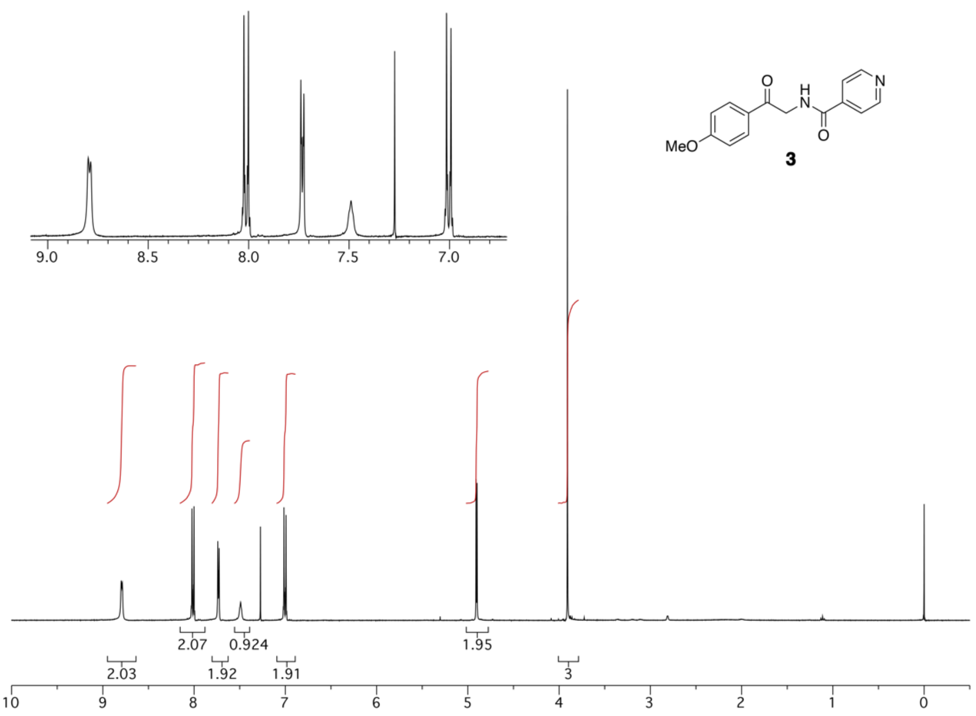

^{13}C NMR ($CDCl_3$, 100 MHz)

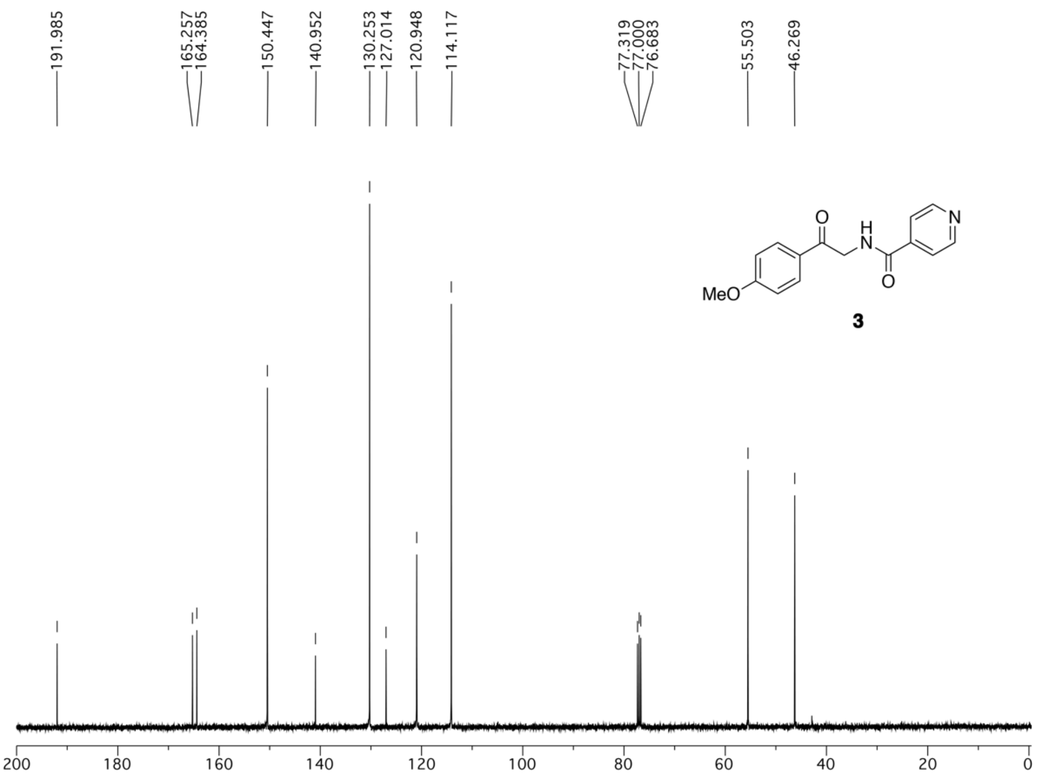

^{1}H NMR (DMSO-d_6, 400 MHz)

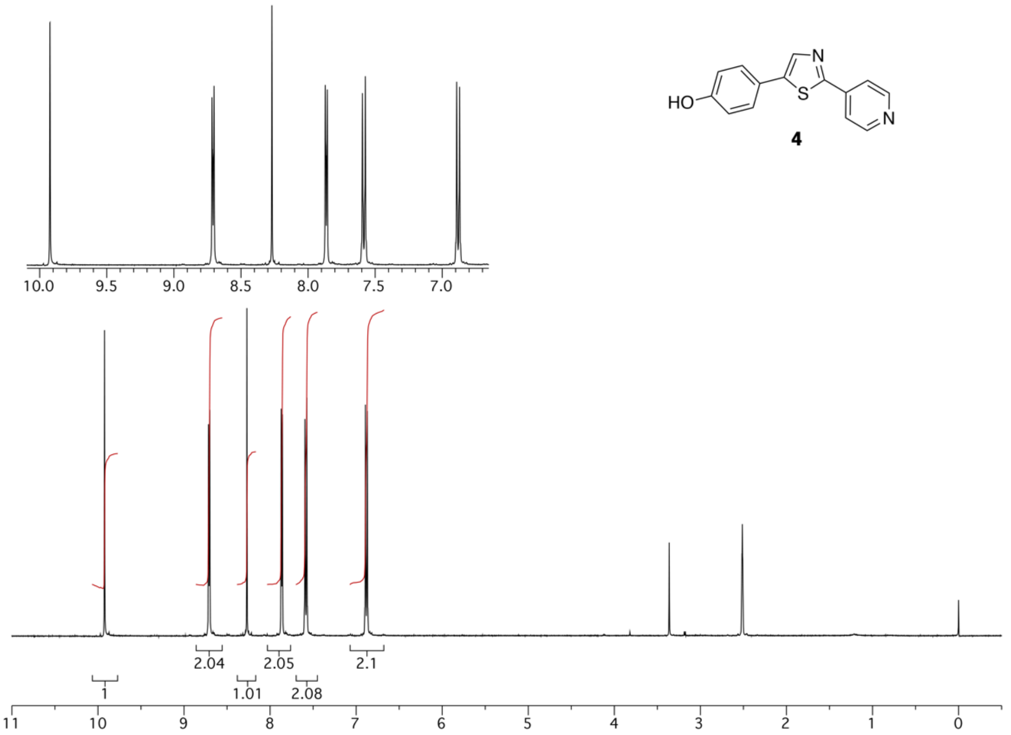

^{13}C NMR (DMSO-d_6, 100 MHz)

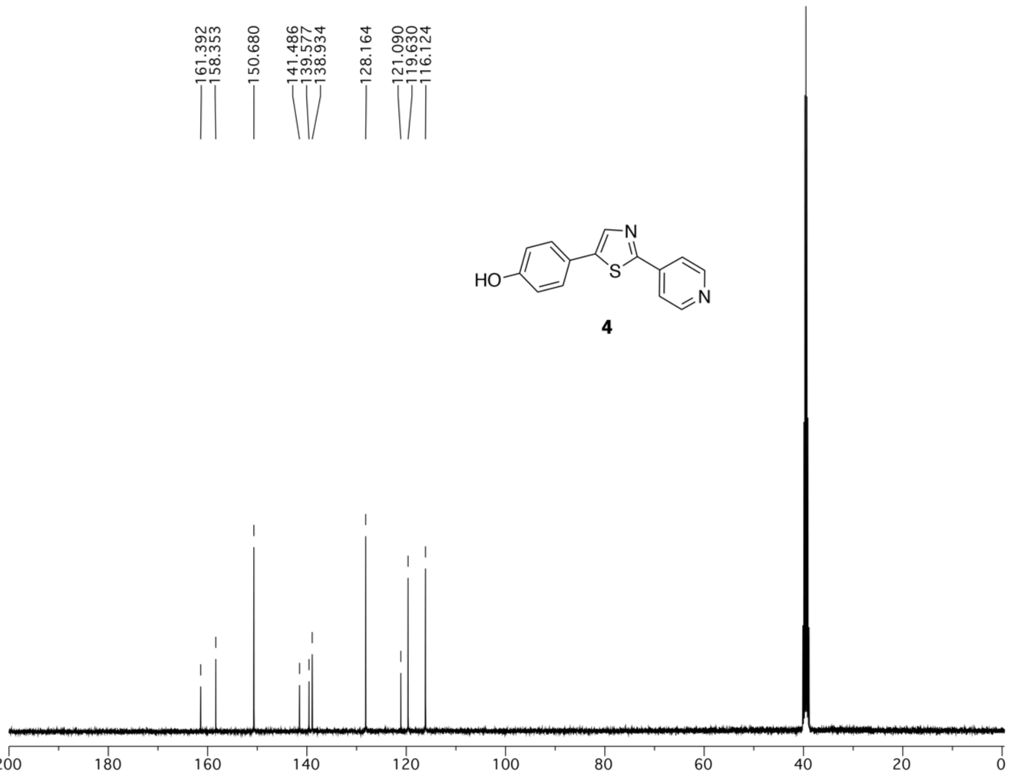

^{1}H NMR ($CDCl_3$, 400 MHz)

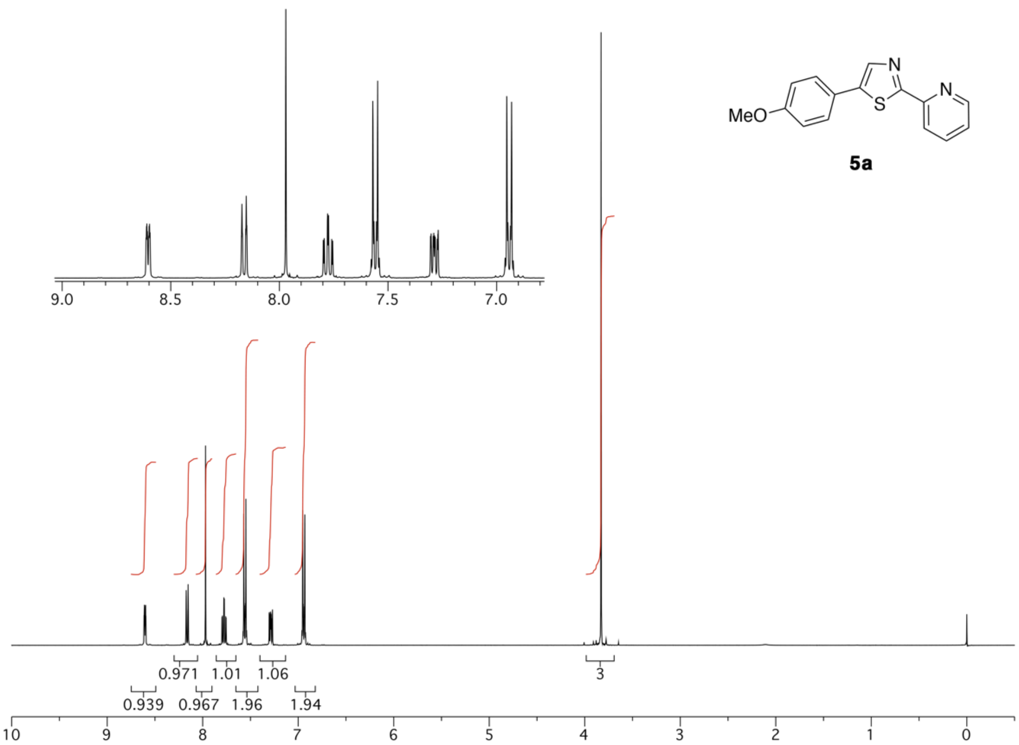

^{13}C NMR ($CDCl_3$, 100 MHz)

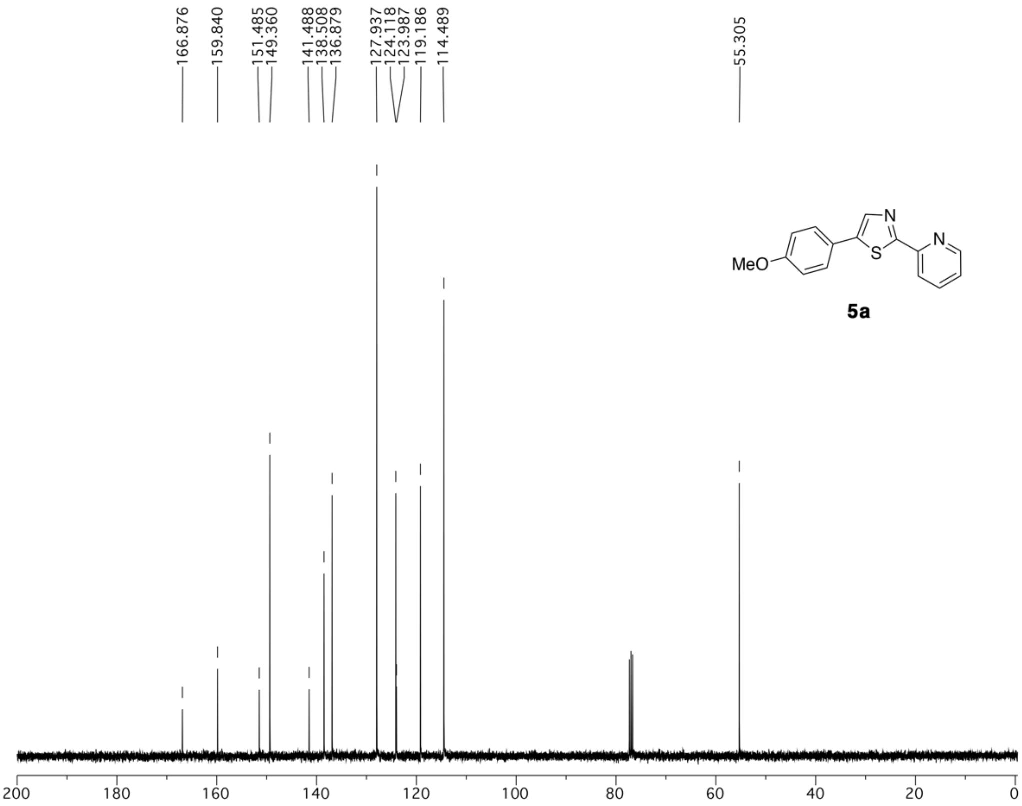

^{1}H NMR (DMSO-d$_6$, 400 MHz)

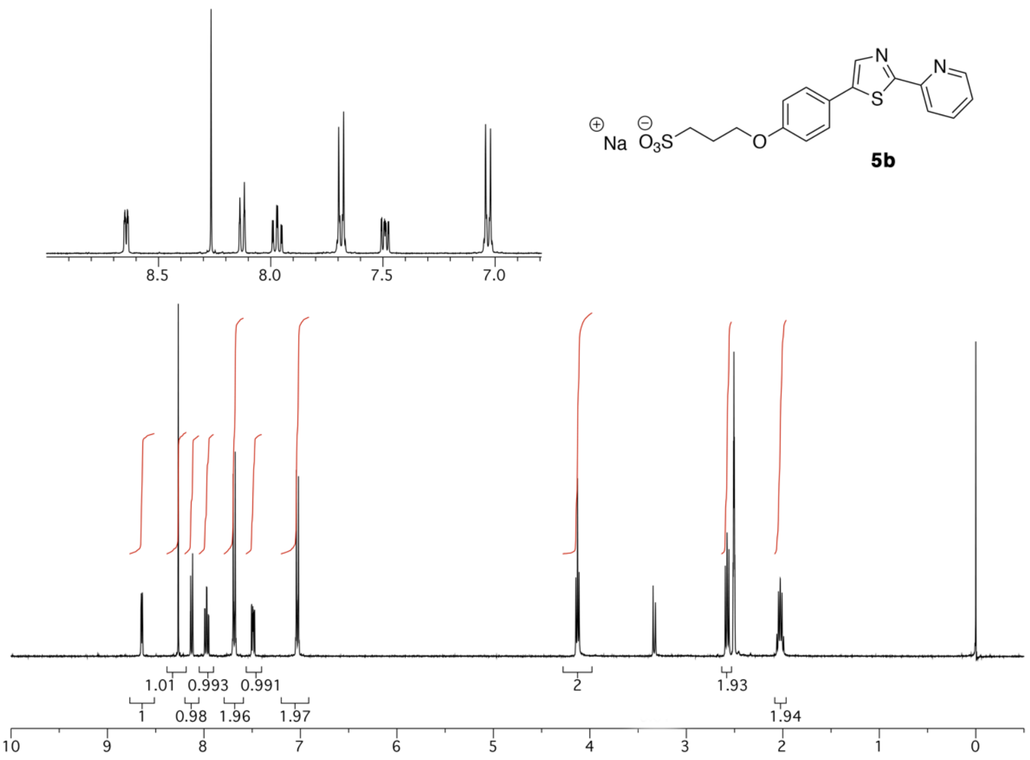

^{13}C NMR (DMSO-d$_6$, 100 MHz)

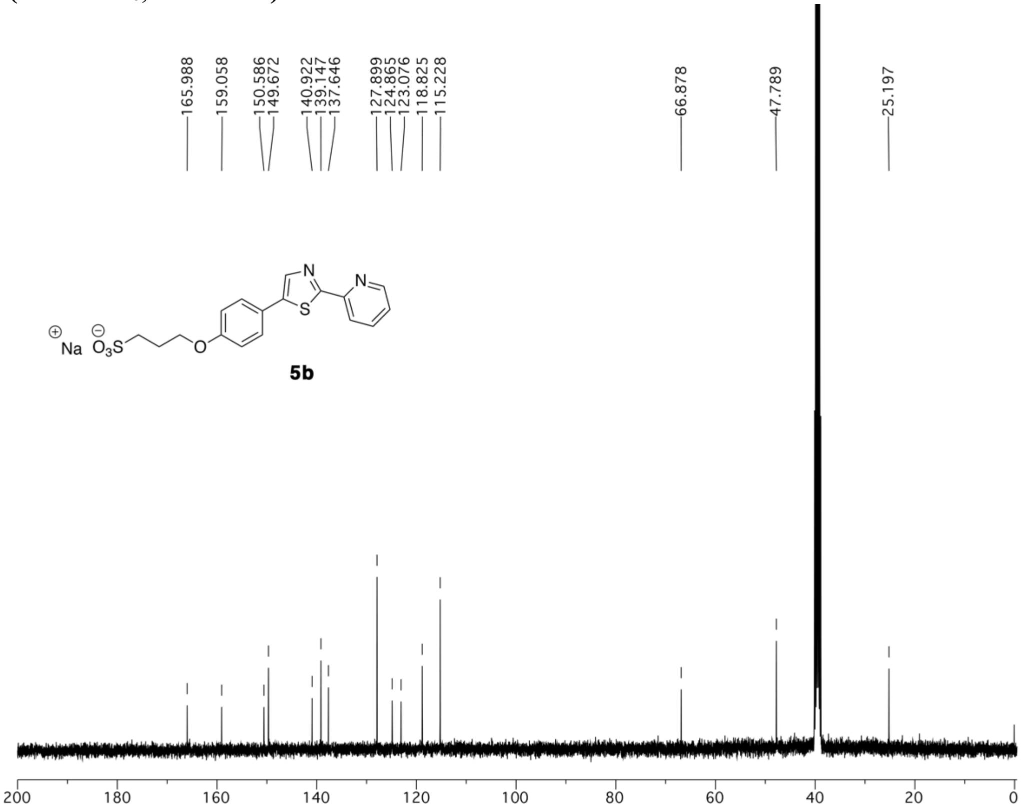

^{1}H NMR ($CDCl_3$, 400 MHz)

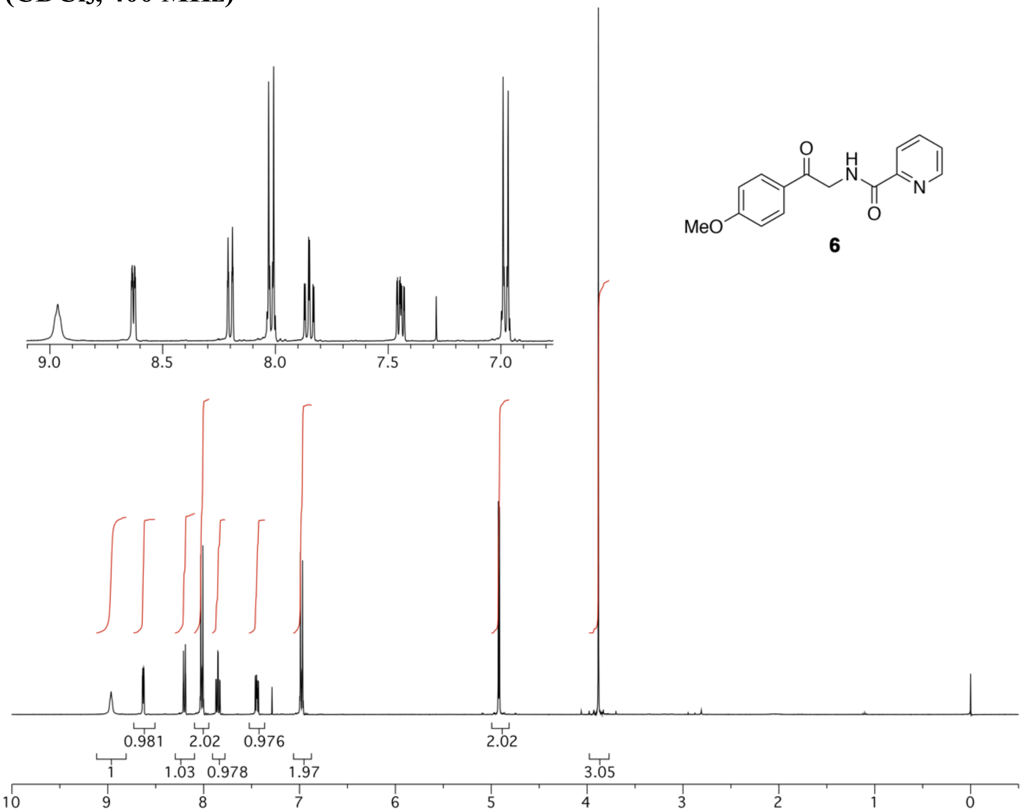

^{13}C NMR ($CDCl_3$, 100 MHz)

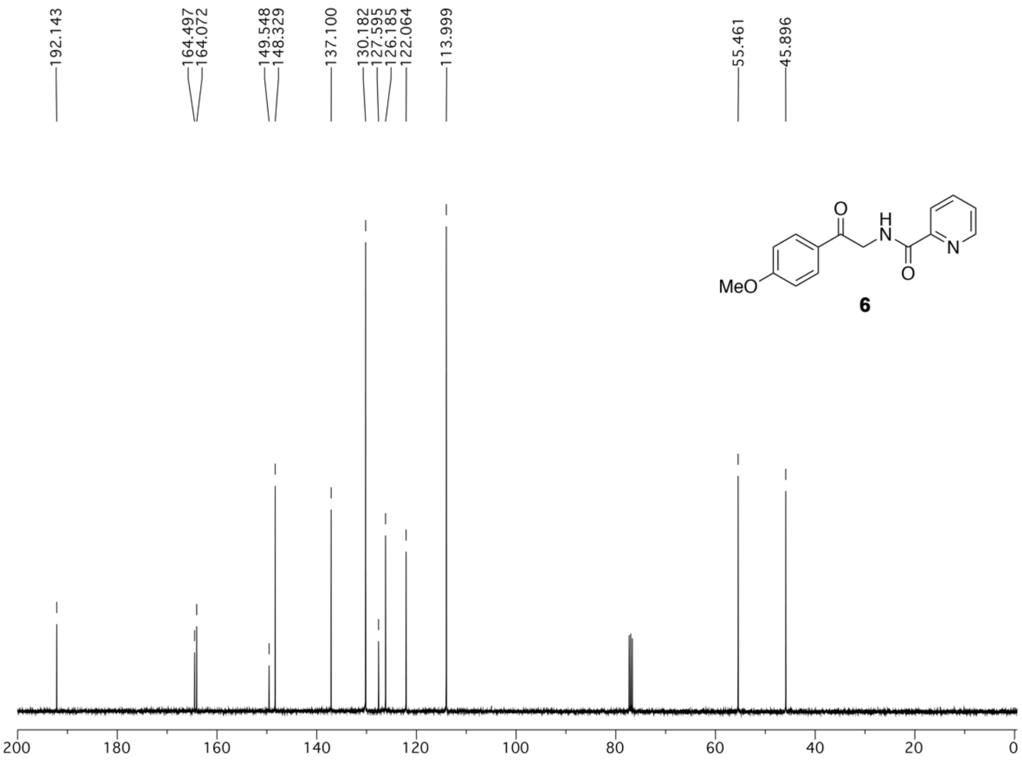

^{1}H NMR (CD_3OD, 400 MHz)

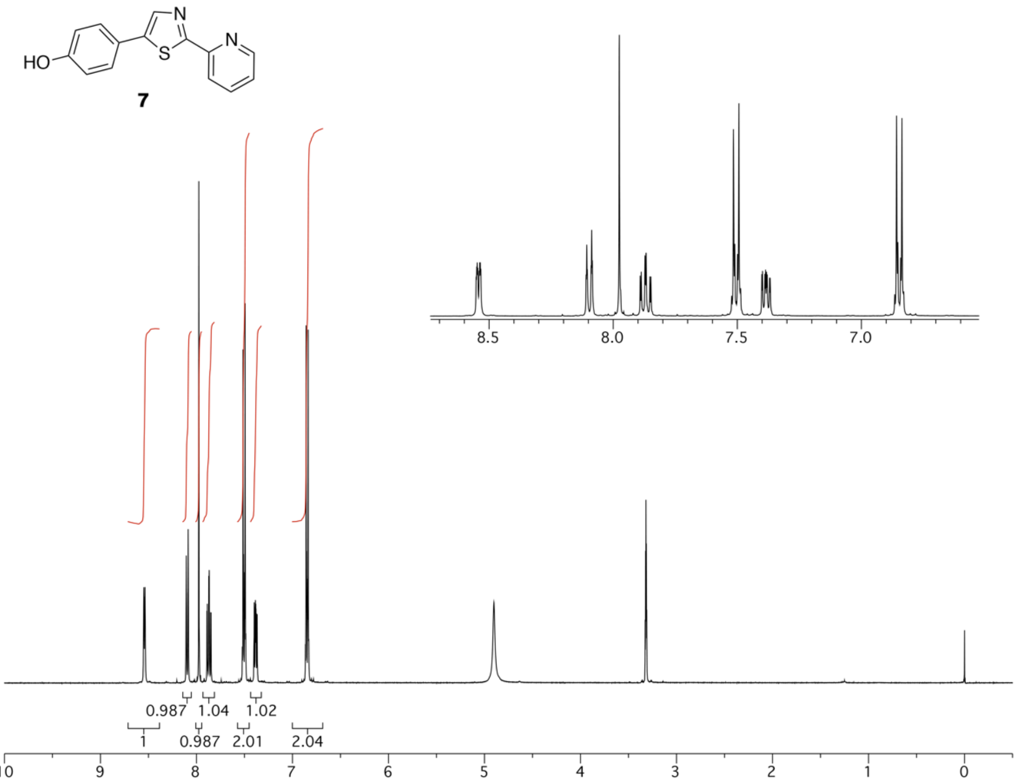

^{13}C NMR (CD_3OD, 100 MHz)

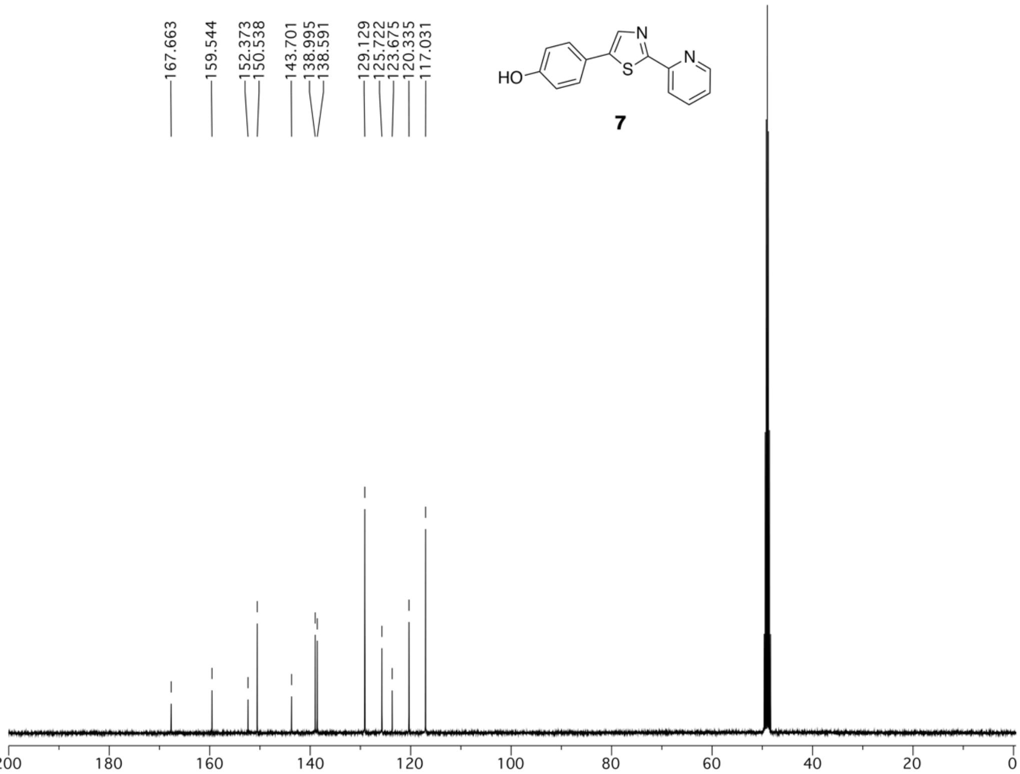

Scheme S1

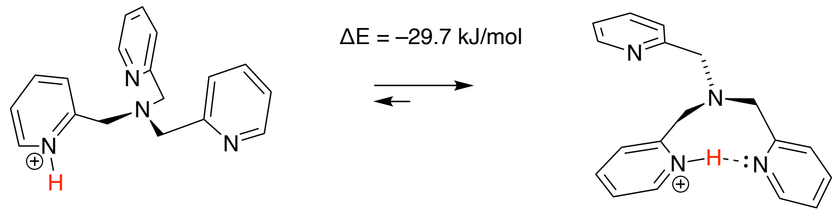

conformer **I** conformer **II**

Molecular geometry of the energy-minimized computational structures (B3LYP/6-31G(d)):

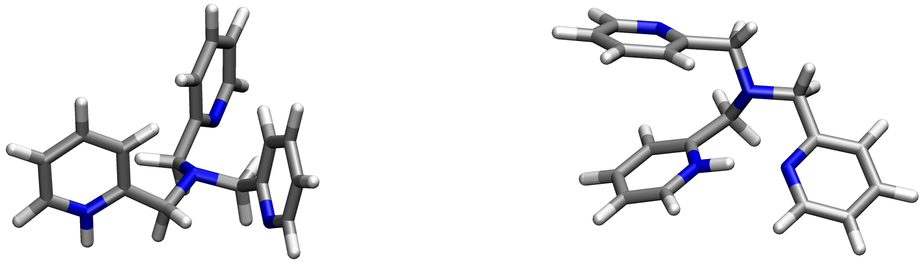

Scheme S2

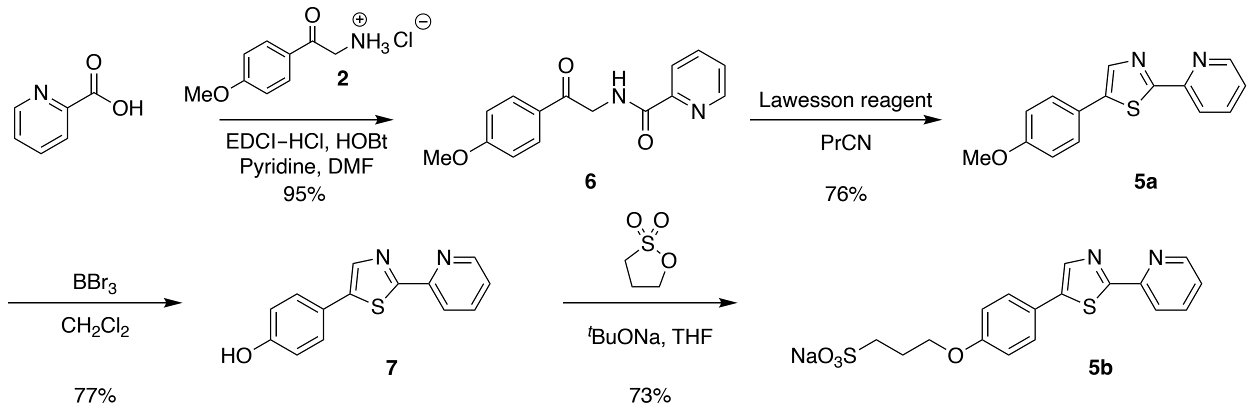

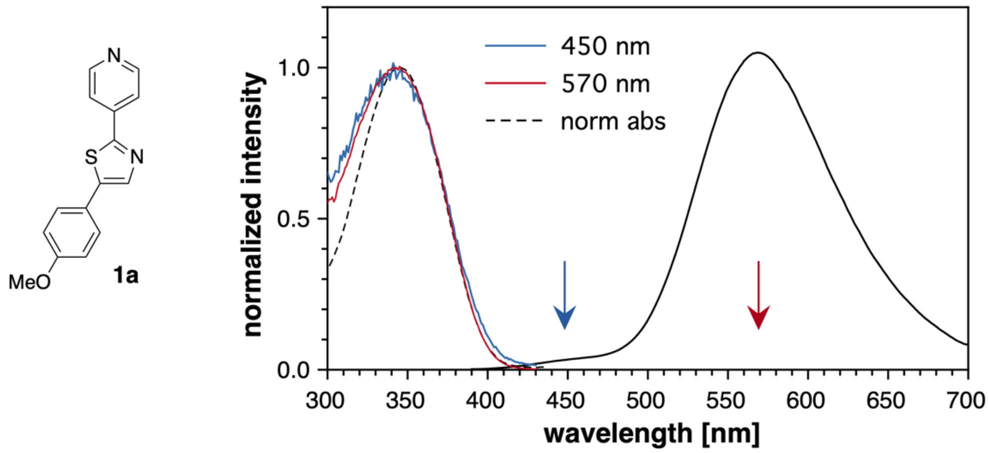

Figure S1. Normalized fluorescence excitation spectra of model fluorophore **1a** in 2,2,2-trifluoroethanol (10 μM, 25°C). The excitation spectra acquired at 450 nm and 570 nm are superimposable and closely match the normalized absorption spectrum of **1a** (dashed trace), indicating that the short and long-wavelength emission originate from the same ground state species. The emission spectrum acquired with excitation at 372 is shown as a solid black trace.

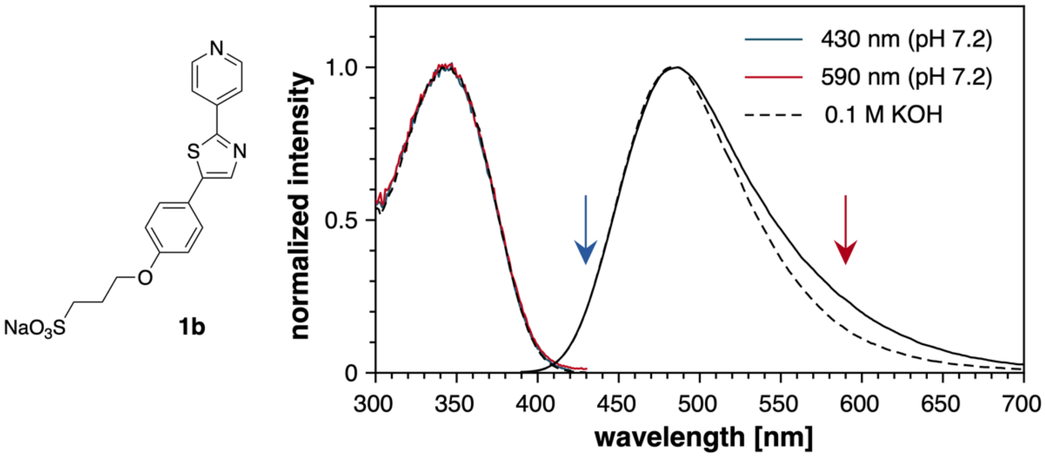

Figure S2. Normalized fluorescence excitation spectra of model fluorophore **1b** in PIPES buffer (pH 7.2, 0.1 M KCl, 25°C). The excitation spectra acquired at the blue (430 nm) and red-edge (590 nm) of the emission spectrum (solid trace) are superimposable and closely match the normalized excitation spectrum in 0.1 M KOH. For comparison, the emission spectrum of **1b** in 0.1 M KOH is shown as a dashed trace.

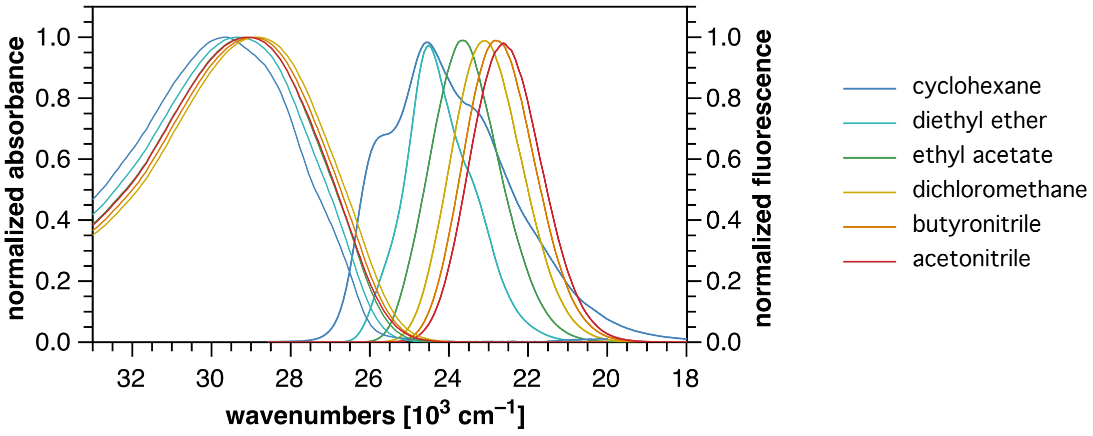

Figure S3. Solvent-dependent solvatochromic shift analysis of fluorophore **1a**. Normalized absorption and fluorescence spectra of model fluorophore **1a** as a function of solvent polarity. The emission spectra are plotted on a wavenumber scale after performing the λ^2 correction. The maximum absorption and emission energies together with the corresponding solvent parameters are compiled in Table S1.

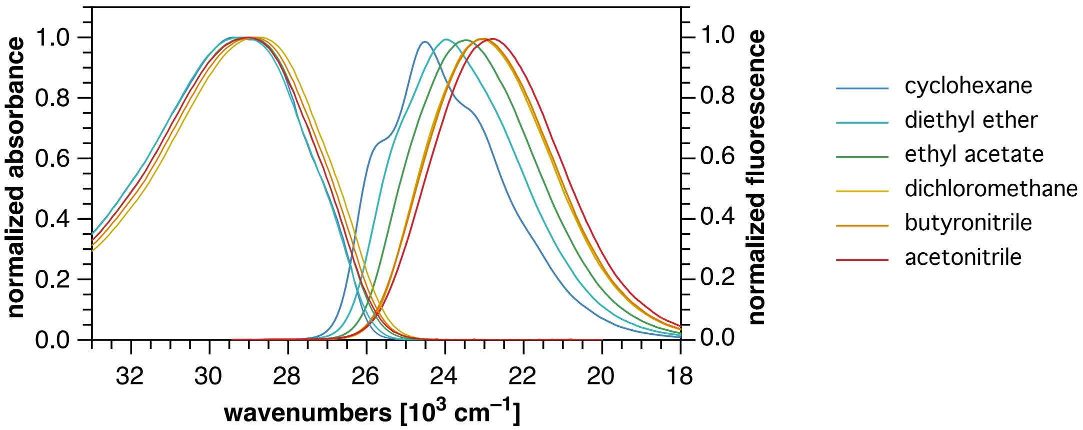

Figure S4. Solvent-dependent solvatochromic shift analysis of fluorophore **5a**. Normalized absorption and fluorescence spectra of model fluorophore **5a** as a function of solvent polarity. The emission spectra are plotted on a wavenumber scale after performing the λ^2 correction. The maximum absorption and emission energies together with the corresponding solvent parameters are compiled in Table S2.

Table S1. Solvent parameters and solvent-dependent absorption and fluorescence emission maxima of model compound **1a**.

Solvent	ε_r	n	$f(\varepsilon_r)$-$f(n)$	ν_{abs} [cm^{-1}]	ν_{em} [cm^{-1}]	ν_{abs}- ν_{em} [cm^{-1}]
Cyclohexane	2.02	1.426	–0.0016	29,673	26,000	3,673
Diethyl ether	4.42	1.352	0.1697	29,327	24,570	4,757
Ethyl acetate	6.053	1.372	0.2004	29,154	23,641	5,513
Dichloromethane	24.56	1.424	0.2668	28,820	23,041	5,779
Butyronitrile	24.56	1.383	0.2809	28,986	22,883	6,103
Acetonitrile	35.94	1.344	0.3046	29,071	22,573	6,498

Table S2. Solvent parameters and solvent-dependent absorption and fluorescence emission maxima of model compound **5a**.

Solvent	ε_r	n	$f(\varepsilon_r)$-$f(n)$	ν_{abs} [cm^{-1}]	ν_{em} [cm^{-1}]	ν_{abs}- ν_{em} [cm^{-1}]
Cyclohexane	2.02	1.426	–0.0016	29,412	24,510	4,902
Diethyl ether	4.42	1.352	0.1697	29,241	24096	5,145
Ethyl acetate	6.053	1.372	0.2004	28,987	23,474	5,513
Dichloromethane	24.56	1.424	0.2668	28,818	23,148	5,670
Butyronitrile	24.56	1.383	0.2809	28,902	22,989	5,913
Acetonitrile	35.94	1.344	0.3046	28,987	22,779	6,208

Table S3. Cartesian atomic coordinates for the geometry-optimized structure of protonated tris(picolyl) amine (Conformer **I**, Scheme S2) with applied PCM correction for water (B3LYP/6-31G(d)). E = –916.207658029 a.u.

Atom	x/Å	y/Å	z/Å
N	-3.506282	-0.939559	-1.153747
N	1.253277	3.030998	-1.135599
N	2.635542	-2.404224	-0.964149
N	-0.083805	-0.044192	-0.787359
C	0.284776	2.386726	-0.461858
C	-2.383246	-0.940986	-0.416421
C	-0.011868	2.665525	0.878971
H	-0.800269	2.115975	1.384386
C	-2.401636	-0.874344	0.983152
H	-1.469047	-0.877271	1.537431
C	1.913174	-1.420574	-0.379788
C	1.862648	-1.393008	1.010620
H	1.283455	-0.612308	1.487212
C	3.283824	-3.351548	1.096832
H	3.822912	-4.112111	1.647731
C	2.547617	-2.355058	1.748920
H	2.512075	-2.329803	2.832929
C	3.313797	-3.356471	-0.283008
H	3.847644	-4.085329	-0.878444
C	-3.628006	-0.807430	1.639605
H	-3.670448	-0.757243	2.723942
C	1.251245	-0.390720	-1.265088
H	1.874795	0.510599	-1.239549
H	1.255791	-0.739251	-2.312978
C	1.946043	3.979534	-0.485261
H	2.722492	4.481368	-1.059807
C	-4.798751	-0.810284	0.879334
H	-5.777285	-0.759618	1.346231
C	1.715433	4.330229	0.843612
H	2.305086	5.109551	1.315852
C	-0.477589	1.314452	-1.213214
H	-1.546351	1.416461	-1.004560
H	-0.333208	1.463169	-2.295035
C	0.712210	3.653893	1.540379
H	0.497391	3.894846	2.577558
C	-1.077334	-1.058001	-1.180143
H	-0.638594	-2.042804	-0.975491
H	-1.302712	-1.022502	-2.258260
C	-4.681094	-0.878795	-0.507744
H	-5.570682	-0.880690	-1.135276
H	2.668361	-2.433037	-1.981300

Table S4. Cartesian atomic coordinates for the geometry-optimized structure of protonated tris(picolyl) amine (Conformer **II**, Scheme S2) with applied PCM correction for water (B3LYP/6-31G(d)). E = –916.218938169 a.u.

Atom	x/Å	y/Å	z/Å
N	2.984741	-1.693468	-0.649070
N	-0.063233	1.511867	0.091957
N	-0.384510	-1.257128	-0.338628
N	-2.562771	0.299165	0.522826
C	0.344980	2.675240	0.631192
C	0.529238	0.939078	-0.978642
C	0.672310	-2.260000	-0.122211
C	1.427777	3.344764	0.086012
C	1.614276	1.582730	-1.561637
C	2.000118	-1.634540	0.263369
C	2.065920	2.789651	-1.026465
C	2.168942	-1.028651	1.515427
C	3.402388	-0.466423	1.833579
C	4.168724	-1.147553	-0.330195
C	4.429515	-0.525685	0.889894
C	-0.083432	-0.358977	-1.459621
C	-1.719263	-1.844072	-0.385376
C	-2.849560	-0.876435	-0.059504
C	-3.574834	1.116542	0.865261
C	-4.170367	-1.258842	-0.327195
C	-4.912366	0.801985	0.656608
C	-5.213094	-0.415059	0.040723
H	0.336237	-2.914433	0.688898
H	0.588537	-0.819452	-2.195471
H	0.835623	-2.884721	-1.015089
H	1.342906	-1.002263	2.219689
H	1.760554	4.277950	0.523677
H	2.103829	1.131890	-2.416881
H	2.917104	3.293363	-1.472738
H	3.561689	0.005445	2.798914
H	4.944704	-1.212572	-1.090797
H	5.408496	-0.102932	1.092285
H	-1.010900	-0.114005	-1.990406
H	-1.750446	-2.641137	0.367012
H	-1.939752	-2.332970	-1.350292
H	-3.289744	2.060532	1.323310
H	-4.368398	-2.209440	-0.813537
H	-5.691702	1.493750	0.957860
H	-6.243313	-0.698383	-0.153340
H	-0.214613	3.034036	1.486042
H	-0.918035	1.009760	0.463954

Table S5. Cartesian atomic coordinates for the geometry-optimized structure of model fluorophore **1a** in the ground state (B3LYP/6-31+G(d)). E = –1161.75593069 a.u.

Atom	x/Å	y/Å	z/Å
S1	-0.769513	-0.732088	-0.174243
N1	-1.691555	1.636114	0.278240
N2	-6.242619	-0.702809	-0.065564
O1	5.997643	-0.014567	-0.151061
C1	4.637308	0.041648	-0.077700
C2	4.054354	1.269406	-0.433077
C3	2.676626	1.426018	-0.397596
C4	1.829183	0.370988	0.005336
C5	2.429587	-0.848282	0.355051
C6	3.814911	-1.022350	0.314621
C7	0.377622	0.566632	0.059909
C8	-0.330130	1.729805	0.281562
C9	-2.095304	0.409009	0.058791
C10	-3.505628	0.006164	0.011474
C11	-4.514946	0.968443	0.184134
C12	-5.847169	0.563670	0.136899
C13	-5.275551	-1.615216	-0.229858
C14	-3.912607	-1.320152	-0.201304
C15	6.650310	-1.233740	0.184364
H1	4.701836	2.082067	-0.748478
H2	2.244856	2.373954	-0.705675
H3	1.811097	-1.678940	0.685441
H4	4.234457	-1.981115	0.597780
H5	0.126649	2.692786	0.480905
H6	-4.251168	2.007016	0.351566
H7	-6.642695	1.294674	0.268770
H8	-5.606797	-2.639137	-0.393098
H9	-3.189849	-2.119411	-0.342275
H10	6.458662	-1.513341	1.228374
H11	7.716306	-1.044965	0.048486
H12	6.335508	-2.049029	-0.479797

Molar volume = 1985.878 $(bohr)^3$/mol (177.218 cm^3/mol)
Recommended a_0 for SCRF calculation = 5.04 Å (9.53 bohr)

Table S6. Cartesian atomic coordinates for the geometry-optimized structure of model fluorophore **1a** in the ground state (B3LYP/6-31+G(d)) with applied SCRF solvent model for water.
E = –1161.76744660 a.u.

Atom	x/Å	y/Å	z/Å
S1	-0.769488	-0.725805	-0.157522
N1	-1.692854	1.646726	0.261542
N2	-6.238890	-0.716529	-0.066922
O1	5.996590	-0.016782	-0.137299
C1	4.636713	0.042906	-0.070278
C2	4.059074	1.287823	-0.375708
C3	2.681091	1.448568	-0.341409
C4	1.828780	0.378260	0.009788
C5	2.423741	-0.858126	0.310813
C6	3.808597	-1.035217	0.271811
C7	0.377324	0.575156	0.061336
C8	-0.330444	1.742818	0.266905
C9	-2.094719	0.413287	0.056555
C10	-3.504236	0.007232	0.010832
C11	-4.520610	0.970407	0.137697
C12	-5.850837	0.560890	0.092653
C13	-5.264294	-1.632110	-0.187795
C14	-3.903928	-1.328635	-0.156474
C15	6.644737	-1.260016	0.149037
H1	4.706750	2.115024	-0.651230
H2	2.258202	2.412939	-0.606813
H3	1.803939	-1.703167	0.599141
H4	4.222905	-2.006492	0.515914
H5	0.125400	2.709162	0.450096
H6	-4.271233	2.017423	0.268334
H7	-6.647361	1.295605	0.189474
H8	-5.585369	-2.663563	-0.315820
H9	-3.179492	-2.131267	-0.259015
H10	6.442139	-1.580950	1.177229
H11	7.711543	-1.069368	0.028359
H12	6.327936	-2.039049	-0.553847

Table S7. Cartesian atomic coordinates for the geometry-optimized structure of model fluorophore **1a** in the lowest-energy excited state (B3LYP/6-31+G(d)). E = –1161.64078971 a.u.

Atom	x/Å	y/Å	z/Å
S1	0.761432	-0.765491	-0.000190
N1	1.688328	1.714648	-0.000050
N2	6.211984	-0.736772	-0.000008
O1	-5.967240	-0.024058	0.000061
C1	-4.617684	0.036187	0.000055
C2	-4.064123	1.340567	0.000105
C3	-2.699791	1.520034	0.000070
C4	-1.799395	0.405612	-0.000016
C5	-2.386434	-0.897299	-0.000090
C6	-3.760883	-1.082023	-0.000060
C7	-0.382869	0.588368	-0.000047
C8	0.356233	1.792715	-0.000062
C9	2.097063	0.421729	-0.000094
C10	3.465039	0.014727	-0.000074
C11	4.506915	0.984921	0.000033
C12	5.826527	0.556915	0.000062
C13	5.225008	-1.653343	-0.000113
C14	3.870229	-1.349152	-0.000147
C15	-6.615970	-1.297063	0.000559
H1	-4.742289	2.188529	0.000183
H2	-2.303718	2.529972	0.000127
H3	-1.740963	-1.770495	-0.000190
H4	-4.159788	-2.090200	-0.000147
H5	-0.105491	2.775475	0.000004
H6	4.260125	2.041201	0.000091
H7	6.632906	1.288625	0.000144
H8	5.545693	-2.694127	-0.000165
H9	3.139373	-2.154344	-0.000233
H10	-6.354343	-1.868844	-0.897773
H11	-7.684263	-1.079456	0.000953
H12	-6.353596	-1.868523	0.898873

Table S8. Cartesian atomic coordinates for the geometry-optimized structure of model fluorophore **1a** in the lowest-energy excited state (B3LYP/6-31+G(d)) with applied SCRF solvent model for water. E = –1161.66647203 a.u.

Atom	x/Å	y/Å	z/Å
S1	0.761110	-0.772612	-0.000282
N1	1.677615	1.708603	-0.000131
N2	6.224532	-0.728869	0.000290
O1	-5.959534	-0.016672	0.000081
C1	-4.618836	0.041613	-0.000078
C2	-4.059660	1.347043	0.000176
C3	-2.697157	1.521962	0.000168
C4	-1.801193	0.398811	-0.000171
C5	-2.394140	-0.905079	-0.000550
C6	-3.765039	-1.084742	-0.000493
C7	-0.391616	0.576243	-0.000157
C8	0.348736	1.786576	-0.000135
C9	2.098080	0.413539	-0.000124
C10	3.461344	0.018602	0.000031
C11	4.510819	0.989292	0.000164
C12	5.829885	0.567746	0.000284
C13	5.232135	-1.648647	0.000118
C14	3.879296	-1.347337	-0.000013
C15	-6.622628	-1.293514	0.000799
H1	-4.732292	2.199092	0.000546
H2	-2.299434	2.530565	0.000569
H3	-1.755422	-1.783012	-0.000937
H4	-4.170115	-2.089597	-0.000795
H5	-0.117355	2.766336	-0.000109
H6	4.272862	2.047401	0.000148
H7	6.630042	1.306155	0.000388
H8	5.549596	-2.690104	0.000084
H9	3.155297	-2.157733	-0.000179
H10	-6.362095	-1.860583	-0.898287
H11	-7.687267	-1.063694	0.001723
H12	-6.360498	-1.860458	0.899498

Table S9. Cartesian atomic coordinates for the geometry-optimized structure of protonated model fluorophore **1a**·HCl in the ground state (B3LYP/6-31+G(d)) with applied SCRF solvent model for water. E = –1622.61027326 a.u.

Atom	x/Å	y/Å	z/Å
Cl1	-8.201807	-0.778364	-0.057267
S1	0.226845	-0.599652	-0.133381
O1	7.011663	-0.270233	-0.138111
N1	-0.555353	1.834917	0.227246
N2	-5.165591	-0.209497	-0.010020
C1	5.659947	-0.134643	-0.075129
C2	5.154775	1.143850	-0.373927
C3	3.789378	1.383035	-0.340117
C4	2.877275	0.358358	0.000284
C5	3.399900	-0.913024	0.294480
C6	4.771136	-1.167430	0.258701
C7	1.442335	0.635248	0.047114
C8	0.801676	1.854082	0.224042
C9	-1.027076	0.616662	0.055593
C10	-2.451041	0.308271	0.029861
C11	-3.392884	1.350916	0.172638
C12	-4.741680	1.062350	0.149011
C13	-4.300550	-1.233584	-0.149959
C14	-2.938723	-1.004995	-0.134244
C15	7.591099	-1.548840	0.146278
H1	5.849463	1.934323	-0.641955
H2	3.423951	2.372242	-0.598508
H3	2.733053	-1.723658	0.576113
H4	5.129594	-2.161519	0.498626
H5	1.315110	2.795775	0.378696
H6	-3.061483	2.373608	0.300476
H7	-5.512313	1.816526	0.253622
H8	-4.734292	-2.218637	-0.271285
H9	-2.270607	-1.850989	-0.248020
H10	7.365702	-1.861457	1.172001
H11	8.666908	-1.414508	0.031428
H12	7.236004	-2.305828	-0.561900
H13	-6.199985	-0.406874	-0.025108

Table S10. Cartesian atomic coordinates for the geometry-optimized structure of model fluorophore **1a**·HCl in the lowest-energy excited state (B3LYP/6-31+G(d)) with applied SCRF solvent model for water. E = –1622.52280825 a.u.

Atom	x/Å	y/Å	z/Å
Cl1	-8.254754	-0.824320	0.000212
S1	0.235745	-0.602554	-0.000103
O1	6.976940	-0.311899	-0.000006
N1	-0.533505	1.906724	0.000244
N2	-5.150671	-0.210603	-0.000171
C1	5.650396	-0.162091	-0.000014
C2	5.184211	1.181550	-0.000232
C3	3.837096	1.449265	-0.000250
C4	2.872504	0.390754	-0.000036
C5	3.366927	-0.949799	0.000164
C6	4.719834	-1.228450	0.000178
C7	1.465774	0.668560	-0.000003
C8	0.811405	1.908957	0.000261
C9	-1.017122	0.650248	-0.000021
C10	-2.415067	0.336480	-0.000107
C11	-3.386507	1.386192	0.000035
C12	-4.725579	1.087375	0.000005
C13	-4.259730	-1.243770	-0.000318
C14	-2.908332	-1.004129	-0.000297
C15	7.562215	-1.629681	0.000188
H1	5.915415	1.983391	-0.000402
H2	3.508808	2.482283	-0.000453
H3	2.667702	-1.780024	0.000335
H4	5.053617	-2.258931	0.000343
H5	1.327937	2.862424	0.000444
H6	-3.067703	2.421333	0.000170
H7	-5.501842	1.843173	0.000104
H8	-4.683248	-2.240687	-0.000457
H9	-2.234716	-1.854533	-0.000421
H10	7.265211	-2.176404	0.899814
H11	8.638004	-1.462432	0.000139
H12	7.265174	-2.176680	-0.899258
H13	-6.169905	-0.415033	-0.000091

Table S11. Cartesian atomic coordinates for the geometry-optimized structure of model fluorophore **5a** in the ground state (B3LYP/6-31+G(d)). E = –1161.76097920 a.u.

Atom	x/Å	y/Å	z/Å
S1	-0.803425	-0.699422	-0.161800
O1	5.972667	-0.043722	-0.153967
N1	-1.698749	1.688740	0.278009
N2	-3.731160	-1.271965	-0.201729
C1	4.611533	0.026815	-0.079574
C2	4.040644	1.258283	-0.440264
C3	2.664172	1.428597	-0.404073
C4	1.805751	0.384860	0.004647
C5	2.395125	-0.838410	0.359506
C6	3.778906	-1.026556	0.318597
C7	0.354949	0.592385	0.059252
C8	-0.335156	1.768207	0.276193
C9	-2.102720	0.460073	0.067173
C10	-3.506562	0.036517	0.018286
C11	-4.544115	0.967894	0.194194
C12	-5.858162	0.512996	0.138099
C13	-6.098739	-0.844863	-0.090150
C14	-4.998575	-1.691457	-0.253022
C15	6.611022	-1.268637	0.185635
H1	4.695790	2.063035	-0.760379
H2	2.241719	2.379377	-0.716478
H3	1.768427	-1.661398	0.693298
H4	4.188664	-1.988441	0.605750
H5	0.132544	2.727736	0.467699
H6	-4.302034	2.010727	0.368422
H7	-6.684111	1.207194	0.270554
H8	-7.108853	-1.240774	-0.141044
H9	-5.140246	-2.755664	-0.432700
H12	6.286156	-2.083271	-0.474654
H10	6.417111	-1.542354	1.230915
H11	7.679240	-1.093290	0.048205

Molar volume = 2027.091 $(bohr)^3$/mol (180.895 cm^3/mol)
Recommended a_0 for SCRF calculation = 5.07 Å (9.59 bohr)

Table S12. Cartesian atomic coordinates for the geometry-optimized structure of model fluorophore **5a** in the ground state (B3LYP/6-31+G(d)) with applied SCRF solvent model for water. E = – 1161.77158519 a.u.

Atom	x/Å	y/Å	z/Å
S1	-0.803478	-0.691386	-0.142999
O1	5.973652	-0.049295	-0.143982
N1	-1.698621	1.701158	0.255873
N2	-3.726561	-1.274679	-0.185916
C1	4.613843	0.025770	-0.074790
C2	4.047868	1.269891	-0.403781
C3	2.671323	1.445247	-0.369019
C4	1.808521	0.391673	0.006105
C5	2.392462	-0.844339	0.329748
C6	3.775655	-1.036519	0.290724
C7	0.358233	0.602881	0.058805
C8	-0.332423	1.781614	0.255774
C9	-2.105841	0.469123	0.062944
C10	-3.509364	0.040105	0.016976
C11	-4.554172	0.964940	0.176567
C12	-5.866871	0.501672	0.122882
C13	-6.098462	-0.860157	-0.087127
C14	-4.993322	-1.703277	-0.235090
C15	6.608924	-1.292974	0.167915
H1	4.702968	2.084876	-0.697829
H2	2.257437	2.408373	-0.652894
H3	1.764778	-1.677027	0.636277
H4	4.180513	-2.007102	0.552997
H5	0.134643	2.744839	0.427923
H6	-4.328415	2.013274	0.337166
H7	-6.696194	1.193041	0.243242
H8	-7.105230	-1.263114	-0.135618
H9	-5.130771	-2.769829	-0.400090
H10	6.404873	-1.589599	1.203124
H11	7.677556	-1.116777	0.041289
H12	6.282309	-2.083675	-0.517249

Table S13. Cartesian atomic coordinates for the geometry-optimized structure of model fluorophore **5a** in the lowest-energy excited state (B3LYP/6-31+G(d)). E = –1161.64602218a.u.

Atom	x/Å	y/Å	z/Å
S1	0.803940	-0.722959	0.000239
O1	-5.937453	-0.058678	-0.000260
N1	1.694872	1.777302	0.000063
N2	3.666169	-1.304463	0.000009
C1	-4.586777	0.018959	-0.000146
C2	-4.049639	1.329141	-0.000153
C3	-2.687027	1.525039	-0.000034
C4	-1.772294	0.422403	0.000115
C5	-2.344248	-0.887002	0.000087
C6	-3.716689	-1.088229	-0.000034
C7	-0.357753	0.618465	0.000269
C8	0.360116	1.837835	0.000139
C9	2.105726	0.487427	0.000087
C10	3.464414	0.051867	0.000001
C11	4.539365	0.980682	-0.000102
C12	5.837750	0.496334	-0.000198
C13	6.048150	-0.895658	-0.000203
C14	4.923180	-1.736399	-0.000095
C15	-6.566550	-1.340174	-0.000006
H1	-4.738021	2.168904	-0.000240
H2	-2.303794	2.540007	-0.000077
H3	-1.688129	-1.752159	0.000170
H4	-4.103303	-2.101230	-0.000085
H5	-0.114117	2.814684	0.000194
H6	4.320569	2.043589	-0.000103
H7	6.680115	1.183648	-0.000291
H8	7.048374	-1.318875	-0.000262
H9	5.052643	-2.818565	-0.000082
H10	-6.296650	-1.909068	-0.897984
H11	-7.638314	-1.139355	0.000094
H12	-6.296421	-1.908823	0.898055

Table S14. Cartesian atomic coordinates for the geometry-optimized structure of model fluorophore **5a** in the lowest-energy excited state (B3LYP/6-31+G(d)) with applied SCRF solvent model for water. E = –1161.66935203 a.u.

Atom	x/Å	y/Å	z/Å
S1	0.796903	-0.742655	0.000038
O1	-5.937769	-0.041193	-0.000001
N1	1.684460	1.756541	-0.000004
N2	3.697988	-1.311384	-0.000012
C1	-4.594683	0.028939	0.000003
C2	-4.046529	1.337734	0.000002
C3	-2.684728	1.524304	0.000005
C4	-1.777825	0.409682	0.000006
C5	-2.361362	-0.898369	0.000002
C6	-3.731776	-1.089086	0.000001
C7	-0.370639	0.596936	0.000016
C8	0.355401	1.819998	0.000019
C9	2.110160	0.463761	-0.000001
C10	3.464387	0.046474	-0.000021
C11	4.535902	0.990183	-0.000049
C12	5.840292	0.527860	-0.000065
C13	6.076884	-0.861246	-0.000054
C14	4.965547	-1.719639	-0.000027
C15	-6.585648	-1.324111	0.000073
H1	-4.725829	2.184643	0.000001
H2	-2.296368	2.536678	0.000009
H3	-1.715741	-1.771294	-0.000001
H4	-4.128335	-2.097436	-0.000007
H5	-0.121204	2.794816	0.000009
H6	4.310315	2.051285	-0.000058
H7	6.670025	1.229913	-0.000087
H8	7.083542	-1.267586	-0.000067
H9	5.116612	-2.798748	-0.000021
H10	-6.318801	-1.889673	-0.898472
H11	-7.653274	-1.107570	0.000140
H12	-6.318680	-1.889634	0.898605
